# Reactions of Heterometallic Phosphinidene-Bridged MoMn and MoRe Complexes with Sulfur and Selenium: From Chalcogenophosphinidene- to Trithiophosphonate-Bridged Derivatives

**DOI:** 10.1021/acs.inorgchem.3c00230

**Published:** 2023-03-29

**Authors:** M. Angeles Alvarez, M. Esther García, Daniel García-Vivó, Miguel A. Ruiz, Patricia Vega

**Affiliations:** Departamento de Química Orgánica e Inorgánica/IUQOEM, Universidad de Oviedo, Oviedo E-33071, Spain

## Abstract

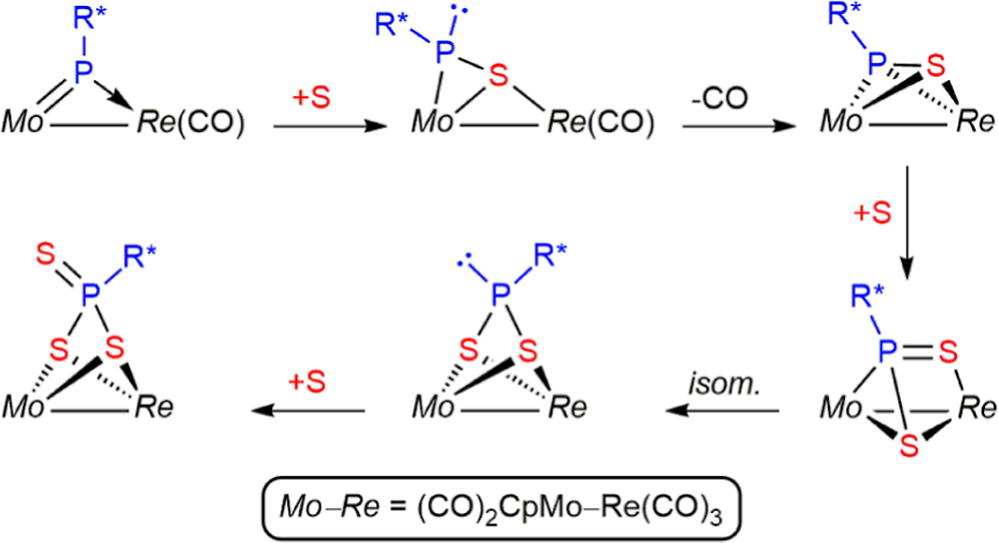

Reactions of [MoReCp(μ-PR*)(CO)_6_] with S_8_ were strongly dependent on experimental conditions (R* = 2,4,6-C_6_H_2_^*t*^Bu_3_). When using 1 equiv of sulfur, complex [MoReCp(μ-η^2^:κ^1^_S_-SPR*)(CO)_6_] was slowly formed at 313 K, with a thiophosphinidene ligand unexpectedly bridging the dimetal center in the novel μ-κ^1^_S_:η^2^ coordination mode, as opposed to the μ-κ^1^_P_:η^2^ mode usually found in related complexes. The latter underwent fast decarbonylation at 363 K to give [MoReCp(μ-η^2^:η^2^-SPR*)(CO)_5_], with a six-electron donor thiophosphinidene ligand rearranged into the rare μ-η^2^:η^2^ coordination mode. Depending on reaction conditions, reactions with excess sulfur involved the addition of two or three S atoms to the phosphinidene ligand to give new complexes identified as the dithiophosphinidene-bridged complex [MoReCp(μ-η^2^:κ^2^_S,S′_-S_2_PR*)(CO)_5_], its dithiophosphonite-bridged isomer [MoReCp(μ-κ^2^_S,S′_:κ^2^_S,S′_-S_2_PR*)(CO)_5_], or the trithiophosphonate-bridged derivative [MoReCp(μ-κ^2^_S,S′_:κ^2^_S,S′_-S_3_PR*)(CO)_5_], all of them displaying novel coordination modes of their PRS_2_ and PRS_3_ ligands, as determined by X-ray diffraction studies. In contrast, the related MoMn complex yielded [MoMnCp(μ-η^2^:η^2^-SPR*)(CO)_5_] under most conditions. A similar output was obtained in reactions with gray selenium for either MoRe or MoMn phosphinidene complexes, which under different conditions only gave the pentacarbonyl complexes [MoMCp(μ-η^2^:η^2^-SePR*)(CO)_5_] (M = Re, Mn), these providing a new coordination mode for selenophosphinidene ligands.

## Abstract 2

Reactions of [MoReCp(μ-PR*)(CO)_6_] with sulfur first resulted in insertion into the Re−P bond followed by decarbonylation to give derivatives with thiophosphinidene ligands in the μ-κ^1^_S_:η^2^ and μ-η^2^:η^2^ coordination modes (R* = 2,4,6-C_6_H_2_^*t*^Bu_3_). Further addition of sulfur yielded a dithiophosphinidene-bridged derivative which rearranged into the corresponding dithiophosphonite-bridged isomer, the latter still adding a third S atom to give the corresponding trithiophosphonate-bridged derivative. All these complexes displayed novel or rare coordination modes of their *P*,*S*-donor ligands.

## Introduction

**Chart 1:**
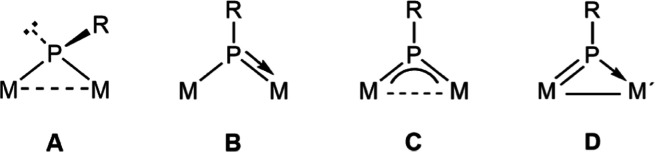
Coordination modes of PR ligands at binuclear complexes.

Mononuclear metal complexes bearing terminal phosphinidene ligands (PR) have been extensively studied as precursors of a great variety of organophosphorus molecules, thanks to their high reactivity toward small organic molecules and other main group compounds.^1,2^ In contrast, only more recently, these studies have been extended to binuclear species having bridging PR ligands to find that the particular coordination mode of the latter (**A** to **C** in Chart 1) greatly influences not only the nature of the new organophosphorus ligands to be formed but also their coordination modes.^3^ Most of this previous work, however, has been carried out using *homometallic* complexes, while we know little yet about the cooperative and synergic effects that the combination of distinct metal atoms with different electron densities and coordination spheres, as found in *heterometallic* complexes,^4^ may induce in the case of phosphinidene-bridged binuclear complexes. Recently, we reported a good-yield synthesis for the heterometallic complex [MoReCp(μ-PR*)(CO)_6_] (R* = 2,4,6-C_6_H_2_^*t*^Bu_3_) (**1a**)^5^ and found that, in spite of the isoelectronic nature of its Mo and Re fragments, the metal-phosphorus π-bonding interaction in this molecule is essentially located at the Mo–P junction to configure a new coordination mode of the bridging phosphinidene ligand (**D** in Chart 1), deserving some studies about the reactivity associated with it. Previous studies on the behavior of **1a** have unveiled a defined tendency of this complex to undergo cycloaddition processes at the Mo–P double bond when reacting with organic molecules having C–C, C–N, and N–N multiple bonds, such as alkynes, isocyanides, diazoalkanes, and organic azides, to build novel or unusual ligands in new coordination modes.^5,6^ This prompted us to further explore the chemical behavior of complexes of type **D**^7^ by examining their reactions with other main group molecules and elements.

**Scheme 1:**
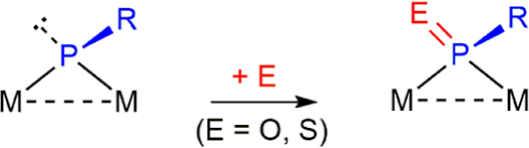
Chalcogen Derivatives of Complexes of Type **A**

In this paper, we analyze the reactivity of **1a** and that of its manganese analogue [MoMnCp(μ-PR*)(CO)_6_] (**1b**)^7^ toward sulfur and selenium. No reaction or full decomposition was observed when these complexes were confronted with tellurium, oxygen, or different O-transfer reagents (olefin oxides, nitric oxide, and peroxo-compounds). Previous studies on reactions of homometallic PR-bridged complexes with chalcogens are scarce, yet they indicate that the coordination mode of the PR ligand, the substituent R, and the metal has a significant influence on the result of these reactions, particularly on the coordination mode of the newly generated organophosphorus ligands. Type **A** (pyramidal) phosphinidene complexes display the most straightforward behavior, that is, the addition of a single chalcogen atom E to the lone pair-bearing P atom, to yield chalcogenophosphinidene ligands bridging the dimetal center in the symmetrical μ-κ^1^_P_:κ^1^_P_ coordination mode, as found in different Pt_2_,^8^ Fe_2_,^9,10^ and Mo_2_ complexes^11^ (Scheme 1, E = O or S). Interestingly, the diplatinum complex [Pt_2_(μ-PMes)_2_(dppe)] (dppe = Ph_2_PCH_2_CH_2_PPh_2_) underwent further addition of S atoms and degradation to eventually yield the mononuclear trithiophosphonate complex [Pt(κ^2^_S,S′_-S_3_PMes)(dppe)].

**Scheme 2:**
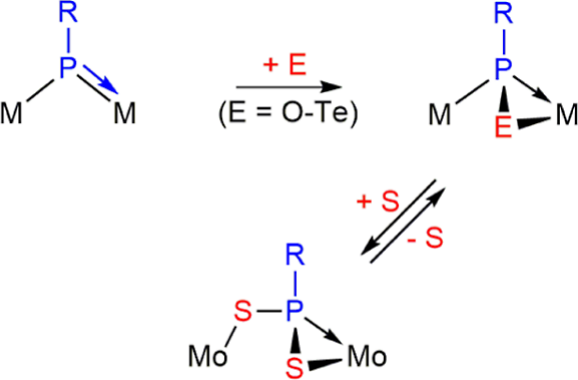
Chalcogen Derivatives of Complexes of Type **B**

Complexes of type **B**, which display trigonal phosphinidene ligands asymmetrically bridging metal fragments of different electron counts (usually 17 and 15 electrons), are instead expected to undergo [2 + 1] cycloaddition of chalcogen atoms to the double M–P bond of these complexes in a plane perpendicular to the M–P–M plane [where the π(M–P) interaction is located]. This should render chalcogenophosphinidene ligands bridging the dimetal center in the asymmetric μ-κ^1^_P_:η^2^ coordination mode, as indeed found in reactions with different homometallic Mo_2_ complexes (Scheme 2, E = O, S, Se, Te)^12^ and heterometallic MoW complexes (E = S, Se).^13^ Remarkably, only one of these complexes ([Mo_2_Cp(μ-κ^1^:κ^1^,η^5^-PC_5_H_4_)(CO)_2_(η^6^-R*H)]) was able to add a second chalcogen atom (S). This yielded a bridging dithiophosphinidene ligand displaying the novel μ-κ^1^_S_:η^2^_P_,_S′_ coordination mode in a process that could be reversed upon reaction with PPh_3_.^12a^

**Scheme 3:**
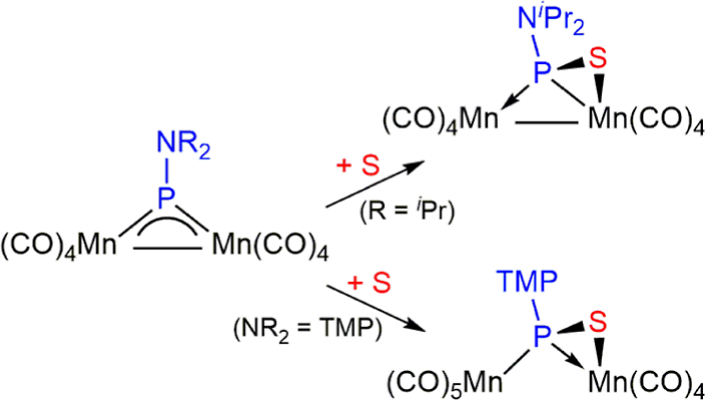
Chalcogen Derivatives of Complexes of Type **C** TMP = tetramethylpiperidyl.

Studies on reactions of chalcogens with complexes of type **C**, which display trigonal phosphinidene ligands bridging symmetrically metal fragments of identical electron counts, have been limited to the dimanganese complexes [Mn_2_(μ-PNR_2_) (CO)_8_] (NR_2_ = TMP, N^*i*^Pr_2_) and a discandium complex, with very different outputs. The Sc_2_ complex reacted with sulfur or selenium by promoting the reductive coupling of two PMes ligands to give chalcogenide- and diphosphene-bridged derivatives.^14^ In contrast, the Mn_2_ complexes reacted with sulfur to give bridging κ^1^_P_:η^2^-thiophosphinidene ligands that displayed a flexible electron contribution to the complex, depending on the electron needs of each metal center (Scheme 3).^15^

With these precedents at hand, it was not obvious at all what the output of the reactions of compounds **1a,b** toward chalcogens would be in terms of both extent in the number of chalcogen atoms adding to the phosphinidene ligand and the coordination mode of the resulting multidentate donor group. As it will be shown below, the heterometallic complexes **1a,b** have shown a behavior more complex than the homometallic complexes mentioned above because, depending on conditions, they are able to add up to three sulfur atoms (but only a selenium one) and undergo different unexpected rearrangements, whereby five new coordination modes of the resulting *P*-and *S*(Se)-donor bridging ligands have been uncovered.

## Results and Discussion

### Stoichiometric Reactions of Compounds **1** with Sulfur

**Scheme 4:**
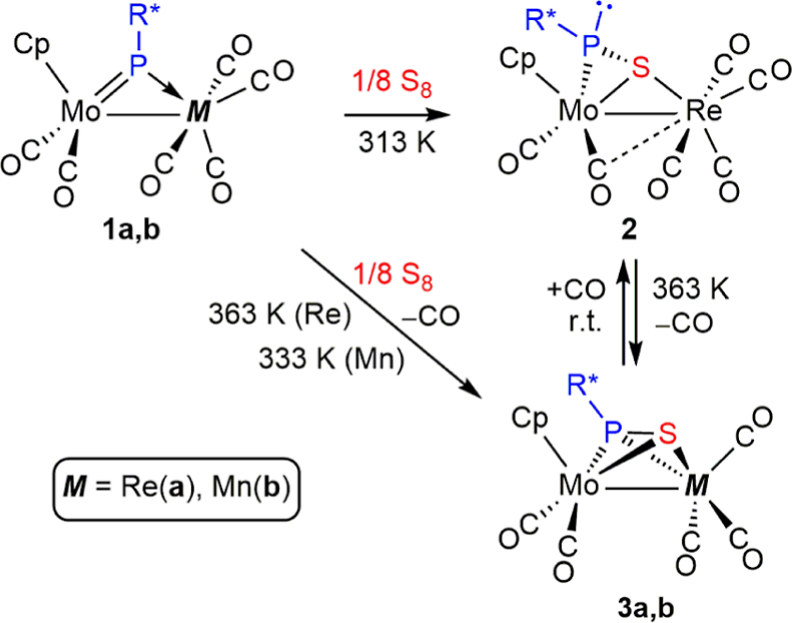
Thiophosphinidene Derivatives of Compounds **1**

Reactions of compounds **1** with S_8_ were strongly dependent on experimental conditions, particularly the relative amount of sulfur used and temperature. Under stoichiometric conditions (1 equiv of sulfur) and upon mild heating (313 K), the rhenium complex **1a** reacted slowly with S_8_ to give the hexacarbonyl complex [MoReCp(μ-η^2^:κ^1^_S_-SPR*) (CO)_6_] (**2**) as a major product, with a thiophosphinidene ligand unexpectedly bridging the dimetal center in the novel μ-κ^1^_S_:η^2^ coordination mode as opposed to the μ-κ^1^_P_:η^2^ mode usually found in reactions of complexes of type **B** or **C** (Schemes 2 and 3). Compound **2** undergoes fast decarbonylation at higher temperatures (363 K) to give cleanly the pentacarbonyl derivative [MoReCp(μ-η^2^:η^2^-SPR*)(CO)_5_] (**3a**), with the thiophosphinidene ligand rearranged into the rare μ-η^2^:η^2^ coordination mode (Scheme 4; see below). The above decarbonylation can be reversed in a few hours upon reaction of **3a** with CO (ca. 4 atm) at room temperature, and this actually provides a more selective preparation of **2** (see the Experimental Section). Expectedly, complex **3a** was the only product formed upon reaction of **1a** with 1 equiv of sulfur at 363 K, thus enabling its isolation as a pure material in 56% yield after crystallization.

Reactions of the manganese complex **1b** with sulfur were much less sensitive to the amount of sulfur added. Indeed only one S atom added to **1b** in its reaction even with excess sulfur (1 equiv. S_8_) if performed at moderate temperatures (333 K), thus giving the thiophosphinidene-bridged complex [MoMnCp(μ-η^2^:η^2^-SPR*)(CO)_5_] (**3b**), which was isolated in 75% yield upon crystallization. In this case, no intermediate species alike compound **2** was detected upon monitoring of this reaction by IR spectroscopy.

### Structure of Thiophosphinidene Complex **2**

**Figure 1:**
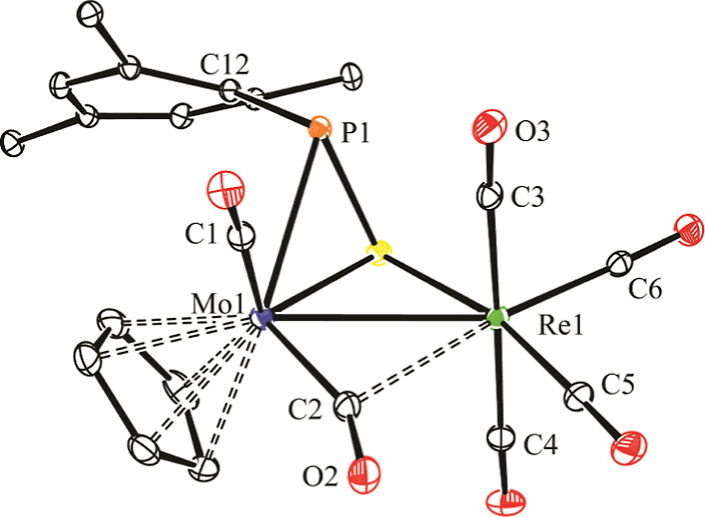
ORTEP diagram (30% probability) of compound **2**, with ^*t*^Bu (except their C^1^ atoms) and H atoms omitted for clarity.

The IR spectrum of **2** displays six C–O stretching bands in the range 2090–1792 cm^–1^ (Table 1); the high frequency and strong intensity of the most energetic band are indicative of the persistence of a disphenoidal Re(CO)_4_ fragment in the molecule.^16^ In addition, the frequency of the less energetic band, mainly arising from the Mo(CO)_2_ fragment, is too low for a terminal carbonyl (1792 cm^–1^), which denotes a semibridging coordination in one of these carbonyl ligands. All of this is consistent with the ^13^C NMR spectrum of the complex, which displays six distinct carbonyl resonances in the range 239–185 ppm, also revealing the absence of any symmetry element in the molecule. An unusual spectroscopic feature of this compound is the large value of the one-bond coupling of the phosphorus nucleus (δ_P_ 31.9 ppm) with the *ipso*-carbon in the aryl ring of the R* group (97 Hz), more commonly found in the range 20–50 Hz (Table 1). Similarly large couplings, which actually are comparable to the couplings measured in phosphorus ylides,^17^ have been previously reported for the phosphapropenediyl-bridged complexes [Mo_2_Cp_2_(μ-κ^1^_C_:η^3^_CCP_-CRCHPR*)(CO)_4_] (R = *p*-tol, CO_2_Me, Pr; ^1^*J*_PC_ = 78–81 Hz)^18^ and are also found in the dithiophosphonite complex **5** (^1^*J*_PC_ = 100 Hz, Table 1). All these molecules feature a lone electron pair at the P atom, a circumstance that might lead to such a strong coupling.^19,20^ This suggests that compound **2** might display a bridging thiophosphinidene ligand bearing a lone electron pair at the P atom as it would occur in the unknown μ-η^2^:κ^1^_S_ coordination mode, a circumstance eventually confirmed through an X-ray diffraction study (Figure 1 and Table 2).

**Table 1:** Selected IR and ^31^P{^1^H} NMR Data for New Compounds.^a^

compound	ν(CO)	δ (P)[^1^*J*_PC_]
[MoReCp(μ-PR*)(CO)_6_] (**1a**)^b^	2077 (m), 1986 (vs), 1951(s), 1876 (w)	673.1[28]
[MoMnCp(μ-PR*)(CO)_6_] (**1b**)^c^	2055 (m), 2039 (w), 1974 (vs), 1951(s), 1862 (w), 1888 (w)	720.9[30]
[MoReCp(μ-η^2^:κ^1^_S_-SPR*)(CO)_6_] (**2**)	2090 (s), 1997 (vs), 1991 (vs, sh), 1955 (s), 1929 (m), 1792 (w)	31.9[97]
[MoReCp(μ-η^2^:η^2^-SPR*)(CO)_5_] (**3a**)	2018 (vs), 1972 (m), 1929 (s), 1906 (m)	–27.7[36]
[MoMnCp(μ-η^2^:η^2^-SPR*)(CO)_5_] (**3b**)	2015 (vs), 1965 (m), 1931 (m), 1909 (m)	23.0[35]
[MoReCp(μ-η^2^:κ^2^_S,S′_-S_2_PR*)(CO)_5_] (**4**)	2027 (vs), 1972 (m), 1929 (m), 1922 (m, sh)	41.4[18]
[MoReCp(μ-κ^2^_S,S′_:κ^2^_S,S′_-S_2_PR*)(CO)_5_] (**5**)	2024 (vs), 1991 (m), 1938 (m), 1916 (m), 1896 (w, sh)	336.2[100]
*syn*-[MoReCp(μ-κ^2^_S,S′_:κ^2^_S,S′_-S_3_PR*)(CO)_5_] (***syn*-6**)	2032 (vs), 1996 (w), 1945 (m), 1926 (m), 1908 (w, sh)	184.0
*anti*-[MoReCp(μ-κ^2^_S,S′_:κ^2^_S,S′_-S_3_PR*)(CO)_5_] (***anti*-6**)	2032 (vs), 1998 (m), 1946 (m), 1925 (m), 1908 (w, sh)	190.5[45]
[MoReCp(μ-η^2^:η^2^-SePR*)(CO)_5_] (**7a**)	2017 (vs), 1971 (m), 1927 (m), 1905 (m)	18.6[35]^d^
[MoMnCp(μ-η^2^:η^2^-SePR*)(CO)_5_] (**7b**)	2013 (vs), 1964 (m), 1930 (m), 1910 (m)	76.1[36]^e^

a IR spectra recorded in dichloromethane solution; ^31^P{^1^H} NMR spectra recorded in CD_2_Cl_2_ solution at 121.48 MHz and 293 K, with chemical shifts (δ) in ppm relative to external 85% aqueous H_3_PO_4_ and coupling constants (*J*) in hertz; ^1^*J*_PC_ data taken from the corresponding ^13^C{^1^H} NMR spectra (see the Experimental Section).

b Data taken from reference (5b).

c Data taken from ref (7).

d *J*(P–^77^Se) = 301.

e *J*(P–^77^Se) = 318.

**Table 2:** Selected Bond Lengths (Å) and Angles (°) for Compound **2**

Mo1–Re1	3.0556(2)	Mo1–S1–Re1	78.00(2)
Mo1–P1	2.6789(7)	P1–Mo1–C1	74.9(1)
Mo1–S1	2.4209(7)	P1–Mo1–C2	120.8(1)
Mo1–C1	1.971(3)	S1–Re1–C3	96.6(1)
Mo1–C2	1.981(3)	S1–Re1–C4	84.6(1)
Re1–S1	2.4346(6)	S1–Re1–C5	168.5(1)
Re1–C3	2.015(3)	S1–Re1–C6	98.4(1)
Re1–C4	2.002(3)	C1–Mo1–C2	80.8(1)
Re1–C5	1.937(3)	C3–Re1–C4	177.1(1)
Re1–C6	1.925(3)	C3–Re1–C5	89.1(1)
Re1···C2	2.542(3)	C3–Re1–C6	90.7(1)
P1–S1	2.091(1)	C5–Re1–C6	91.5(1)
P1–C12	1.861(2)	Mo1–C2–O2	156.6(2)

The molecule of **2** can be derived from that of parent complex **1a**(5) upon full insertion of a sulfur atom into the Re–P bond of the latter complex, with further coordination of this S atom to molybdenum in a rather symmetrical way (Mo–S = 2.4209(7), Re–S = 2.4346(6) Å). This renders a thiophosphinidene ligand bound to the dimetal center in the novel μ-κ^1^_S_:η^2^ coordination mode as suspected,^21^ with a strong pyramidalization at the P atom (Σ(X–P–Y) = 264.0°), which then can be assumed to bear a lone electron pair and which displays quite a large Mo–P separation of 2.6789(7) Å, likely due to the interelectronic repulsion induced by this lone electron pair. In all, the thiophosphinidene ligand provides the dimetal center with four electrons to yield a 34-electron complex for which a metal–metal single bond must be proposed according to the 18-electron rule. This is consistent with the intermetallic length of 3.0556(2) Å, ca. 0.1 Å shorter than the one measured in parent complex (3.1745(6) Å). We finally note that one of the Mo-bound carbonyls is involved in a semibridging interaction with the Re atom [C2···Re1 = 2.542(3) Å, Mo1–C2–O2 = 156.6(2)°], as anticipated by the IR data discussed above, and that the P–S distance of 2.091(1) Å is a bit shorter than the reference value of ca. 2.12 Å for a single bond between these atoms,^22^ which is indicative of the retention of only a modest multiplicity in that bond of the thiophosphinidene ligand.

### Structure of Thiophosphinidene Complexes **3**

**Figure 2:**
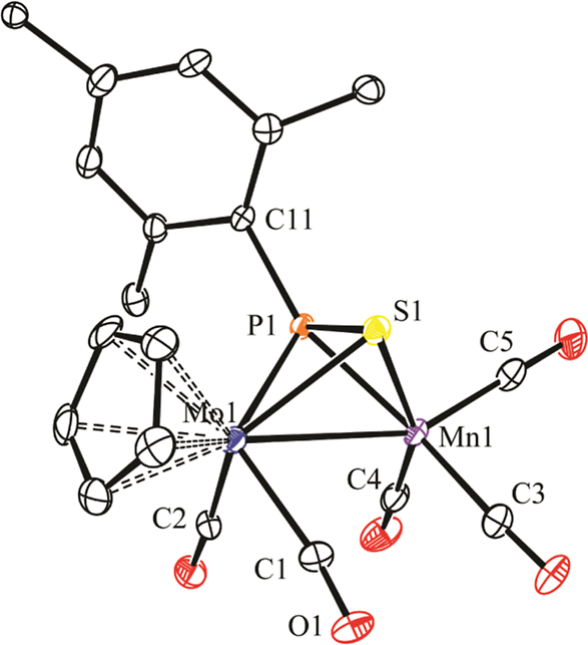
ORTEP diagram (30% probability) of compound **3b**, with ^*t*^Bu (except their C^1^ atoms) and H atoms omitted for clarity. Only one of the two independent molecules in the unit cell shown.

In the crystal, the manganese complex **3b** displays two independent molecules in the asymmetric unit, otherwise similar to each other (Figure 2 and Table 3). The molecule of **3b** can be derived from that of parent compound **1b**(7) after removal of a Mn-bound carbonyl and addition of a sulfur atom to its MoPMn triangle, thus building a tetrahedral MoMnPS central core. As a result, the coordination environment around the Mo atom is one of the classical four-legged piano stool type, while the one around the Mn atom is square-pyramidal if we ignore the intermetallic interaction. In all, the η^2^:η^2^-bound SPR* ligand can be viewed as providing the dimetal center with six electrons (two lone pairs from P and S atoms, added to the π-bonding electrons of the P=S double bond), and therefore, a Mo–Mn single bond must be proposed for this 34-electron complex according to the 18-electron rule. This is consistent with the short intermetallic distance of 3.016(1) Å, actually ca. 0.1 Å shorter than the one measured in the parent complex [3.1049(3) Å]. In all, the structure of **3b** is comparable to the one recently determined by us for the phosphanide- and thiolate-bridged complex [MoReCp(μ-PCy_2_)(μ-SPh)(CO)_5_],^22^ although the intermetallic distance in the latter [2.9702(8) Å] must be considered significantly shorter if we allow for the ca. 0.12 Å difference between the covalent radii of Re and Mn atoms.^23^

**Table 3:** Selected Bond Lengths (Å) and Angles (°) for Compound **3b**

Mo1–Mn1	3.016(1)	Mo1–P1–Mn1	80.46(7)
Mo1–P1	2.447(2)	Mo1–S1–Mn1	77.00(6)
Mo1–S1	2.475(2)	P1–Mo1–C1	118.5(2)
Mo1–C1	2.021(8)	P1–Mo1–C2	88.5(2)
Mo1–C2	1.995(7)	P1–Mn1–C3	153.6(2)
Mn1–P1	2.214(2)	P1–Mn1–C4	104.8(2)
Mn1–S1	2.367(2)	P1–Mn1–C5	104.7(3)
Mn1–C3	1.838(8)	C1–Mo1–C2	81.5(3)
Mn1–C4	1.789(8)	C3–Mn1–C4	95.5(3)
Mn1–C5	1.778(10)	C3–Mn1–C5	91.2(4)
P1–S1	2.074(2)		
P1–C11	1.833(7)		

As for the bridging thiophosphinidene ligand in **3b**, we note that the 36-electron dichromium complex [Cr_2_Cp*_2_(μ-η^2^:η^2^-SPC_6_H_5_OMe)] appears to be the only other complex structurally characterized to date as displaying η^2^:η^2^ coordination of a bridging thiophosphinidene ligand, although the latter configures a butterfly (rather than tetrahedral) Cr_2_PS central core since no metal–metal bond is present. The P–S distance of 2.031(2) Å in that complex is a bit shorter than the one in **3b** (2.074(2) Å), which points to a stronger interaction of the P=S bond with the dimetal center in our case. Yet, the latter distance still falls below the reference value of 2.12 Å for a P–S single bond, which suggests the retention of some multiplicity in that bond of **3b**, as also found for different Mo_2_ complexes displaying μ-κ^1^_P_:η^2^-SPR ligands.^12^ Moreover, since the electron counts of the MoCp(CO)_2_ and Mn(CO)_3_ fragments in **3b** are different from each other (15 and 13 electrons, respectively), we would expect the bridging thiophosphinidene ligand to bind the manganese fragment more tightly, so as to balance this difference. Indeed, the P–Mn distance of 2.214(2) Å in **3b** is significantly shorter than the Mo–P one (2.447(2) Å), even after allowing for the ca. 0.15 Å difference in the covalent radii of these atoms. This difference, however, does not apply to the M–S distances [2.475(2) and 2.367(2) Å] that can be viewed as comparable to each other, under similar terms.

Spectroscopic data in solution for compounds **3a** and **3b** (Table 1 and Experimental Section) are similar to each other and consistent with the solid-state structure just discussed. Their IR spectra display in each case four C–O stretches, with the most energetic one (at ca. 2015 cm^–1^) being of high intensity and of much lower frequency than the corresponding band in either complexes **1** or **2**, thus revealing the presence of a pyramidal M(CO)_3_ fragment in complexes **3a,b**. The corresponding carbonyls gave rise to a single and broad resonance in the respective ^13^C NMR spectra at room temperature, which is indicative of fast rotational exchange on the NMR time scale, a common feature of molecules bearing pyramidal M(CO)_3_ fragments, not further investigated. In contrast, the Mo-bound carbonyls give rise to separate resonances with distinct two-bond P–C couplings, as expected after considering their different positions relative to the P atom [P–Mo–C = 88.5(2), and 118.5(2)° in the crystal].^25^ We finally note the significant shielding of ca. 60 ppm for the ^31^P nucleus in **3a** (δ_P_ −27.7 ppm) when compared to the one in **2** (δ_P_ 32.1 ppm), a difference hard to anticipate on qualitative grounds. In contrast, the fact that the ^31^P NMR resonance of **3a** appears some 45 ppm below the one of its manganese analogue **3b** reflects a shielding effect expected when replacing a transition metal atom with a heavier one within the same group.^26^

### Reactivity of Compounds **1** with Excess Sulfur

**Scheme 5:**
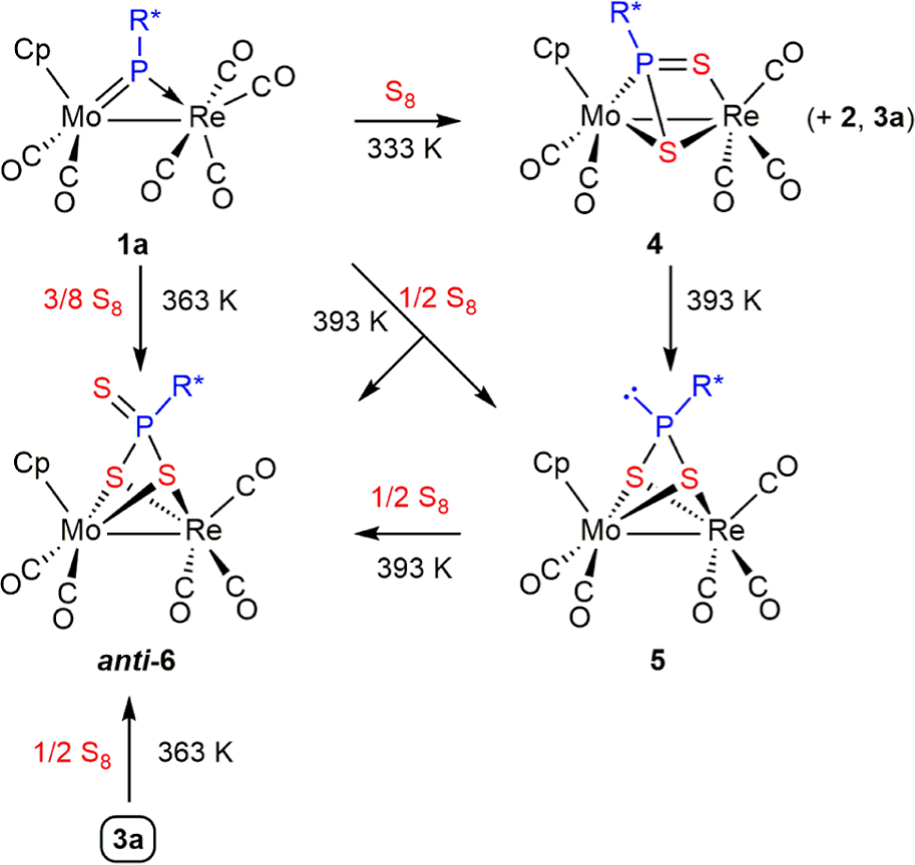
Dithio- and Trithioderivatives of Compound **1a**

Reactions of the rhenium complex **1a** with excess sulfur were of modest selectivity. Depending on the relative amount of sulfur and temperature, they involved the addition of two or three S atoms to the phosphinidene ligand to give as major products new complexes identified as the dithiophosphinidene-bridged complex [MoReCp(μ-η^2^:κ^2^_S,S′_-S_2_PR*) (CO)_5_] (**4**), its dithiophosphonite-bridged isomer [MoReCp(μ-κ^2^_S,S′_:κ^2^_S,S′_-S_2_PR*) (CO)_5_] (**5**), and the anti-isomer of the trithiophosphonate-bridged derivative [MoReCp(μ-κ^2^_S,S′_:κ^2^_S,S′_-S_3_PR*) (CO)_5_] (**6**) (Scheme 5). Fortunately, the above complexes could be satisfactorily purified through chromatographic workup or crystallization and were then fully characterized both in solution and in the solid state.

Dithiophosphinidene complex **4** was better prepared by carrying out the reaction at moderate temperature (333 K) and using 1 equiv of S_8_, although significant amounts of thiophosphinidene complexes **2** and **3a** were also present in the reaction mixture. Actually, compound **4** can be viewed as resulting from insertion of an S atom into the P–Re bond of **3a**. A separate experiment revealed that pure complex **4** rearranged rapidly (30 min) into its dithiophosphonite isomer **5** in refluxing toluene solution, in a process formally involving the reduction of the ligand and oxidation of the dimetal center (see below). However, the direct reaction of **1a** with sulfur (4 equiv) in refluxing toluene did not yield **5** as the sole product but rather a 1:1 mixture of **5** and the trithiophosphonate complex ***anti*-6** after 20 min. This might be considered as expected since **6** is likely to be formed upon addition of an extra sulfur atom to the lone-pair-bearing P atom of **5**. However, an independent experiment indicated that such a process is not fast enough to justify the above observation, as the reaction of **5** with 1/2 equiv S_8_ in refluxing toluene, although indeed gave ***anti*-6** as the major product, required about 3 h for completion. Then, we are bound to conclude that compound ***anti*-6** stems from more than one reaction path. A further independent experiment indicated that thiophosphinidene complex **3a** reacted with 1/2 equiv S_8_ in toluene at 363 K to give ***anti*-6** as the major product in ca. 6 h without detectable intermediates. This prompted us to examine the formation of the latter trithiophosphonate complex at that temperature and found that the reaction of **1a** with 3/8 equiv S_8_ at 363 K was the most selective method to prepare that complex. In all, we conclude that the stepwise sulfurization of the phosphinidene ligand likely is initiated in all cases via thiophosphinidene complexes **2** and **3a** and then go on through two alternative paths: (a) a slower one involving the stepwise formation of **4** (insertion of a second S atom into the Re–P bond), **5** (rearrangement), and **6** (addition of a third S atom to the P atom) and (b) a faster one connecting **3a** with **6** without detectable intermediate species. An attractive hypothesis for the faster route is that it might be initiated by insertion of the second S atom into the Mo–P (instead of Re–P) bond, but other possibilities exist, for instance, addition of the second S atom to one of the metal centers. Unfortunately, we have no data to currently favor one or other alternative routes.

**Scheme 6:**
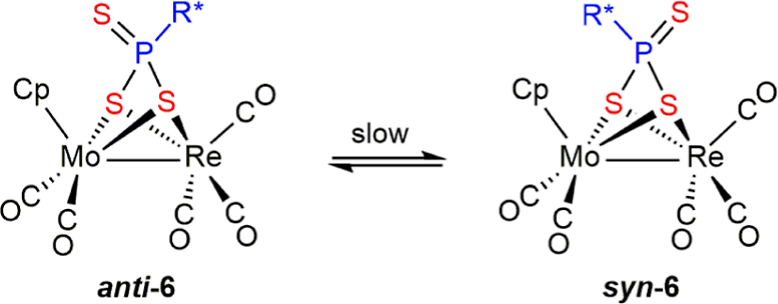
Isomerization in Complexes **6**

As indicated by the crystallographic data to be discussed below, both **5** and ***anti*-6** have the same stereochemistry of the bridging ligand, with the bulky 2,4,6-C_6_H_2_^*t*^Bu_3_ group pointing away from the Cp ligand bound to the Mo atom (*anti* conformation), which is likely the most favored conformation to minimize steric repulsions. We found, however, that ***anti*-6** slowly rearranges in dichloromethane solution to reach a ca. 1:1 equilibrium mixture with its *syn* isomer, the latter having now the bulky aryl group pointing toward the Cp ligand (Scheme 6). Complex ***syn*-6** could be isolated from the above mixtures as a pure solid, and it was fully characterized. Moreover, we found that dissolving this complex in toluene would progressively regenerate the *anti* isomer. From the above experimental data, it can be concluded that both *syn* and *anti* isomers of **6** must be of similar energy and that the appearance of the *syn* isomer in the more polar solvent might be due to its higher dipolar moment, which would render more favorable dipole–dipole interactions with the solvent. These conclusions were supported by density functional theory (DFT) calculations on both isomers (see the Experimental Section and the Supporting Information), which yielded for ***anti*-6** a Gibbs free energy at 298 K just 9 kJ/mol below that of ***syn*-6**, while the latter complex would have a notably higher dipole moment (4.0 and 11.6 D, respectively).

Reactions of the manganese complex **1b** with excess sulfur yielded mixtures of complexes that could not be properly isolated nor fully characterized. Among these, we could only identify the trithiophosphonate complex *anti*-[MoMnCp(μ-κ^2^_S,S′_:κ^2^_S,S′_-S_3_PR*) (CO)_5_] on the basis of similarities with the spectroscopic data of ***anti*-6**,^27^ but we could not isolate it as a pure material, and then it was not further investigated.

### Structure of Dithiophosphinidene Complex **4**

**Figure 3:**
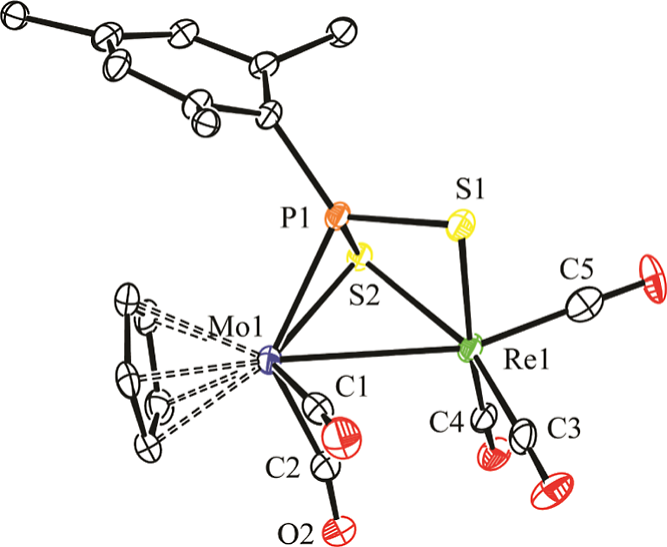
ORTEP diagram (30% probability) of compound **4**, with ^*t*^Bu (except their C^1^ atoms) and H atoms omitted for clarity.

**Chart 2:**
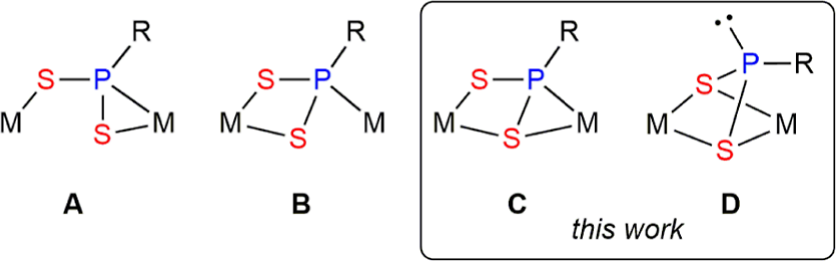
Coordination modes of bridging PRS_2_ ligands.

The molecule of this complex in the crystal (Figure 3 and Table 4) can be derived from that of thiophosphinidene complex **3b** by replacing in the latter the manganese atom with rhenium and inserting a sulfur atom into the corresponding Re–P bond, while the former S atom remains bound to both metal atoms. This results in a dithiophosphinidene ligand bridging the dimetal center in the novel μ-η^2^:κ^2^_S,S′_ coordination mode (**C** in Chart 2), while the square pyramidal environment around the group 7 metal atom is retained. We note that there are only a few binuclear complexes with bridging PRS_2_ ligands structurally characterized previously, these displaying either μ-η^2^:κ^1^_S_ (**A**, 4-electron donor)^12a^ or μ-κ^1^_P_:κ^2^_S,S′_ (**B**, 6-electron donor, Chart 2) coordination modes.^24,28^ In complex **4**, the PRS_2_ ligand can be viewed as providing the dimetal center with six electrons (two lone pairs from S atoms, added to the π-bonding electrons of a P=S double bond), which makes it electron-precise (34 electron). Therefore, a Mo–Re single bond must be proposed for this molecule according to the 18-electron rule, which is consistent with the intermetallic distance of 3.0693(4) Å, somewhat shorter than the one measured in parent complex **1a** [3.1745(6) Å], but to be considered only a bit shorter than the one measured in the manganese complex **3b** [3.016(1) Å] if we allow for the different covalent radii of Mn and Re atoms.^22^

**Table 4:** Selected Bond Lengths (Å) and Angles (°) for Compound **4**

Mo1–Re1	3.0693(4)	Mo1–S2–Re1	76.32(3)
Mo1–P1	2.481(1)	Re1–S1–P1	80.39(5)
Mo1–S2	2.502(1)	P1–Mo1–C1	86.1(2)
Mo1–C1	1.982(6)	P1–Mo1–C2	129.7(2)
Mo1–C2	2.019(6)	S1–Re1–C3	95.1(2)
Re1–S1	2.524(1)	S1–Re1–C4	169.2(2)
Re1–S2	2.465(1)	S1–Re1–C5	86.4(2)
Re1–C3	1.932(6)	C1–Mo1–C2	83.9(2)
Re1–C4	1.917(5)	C3–Re1–C4	93.1(2)
Re1–C5	1.908(7)	C3–Re1–C5	89.4(2)
P1–S1	2.019(2)	C11–P1–Mo1	120.1(2)
P1–S2	2.093(2)	C11–P1–S1	123.2(2)
P1–C11	1.838(5)	C11–P1–S2	111.3(2)

**Chart 3:**
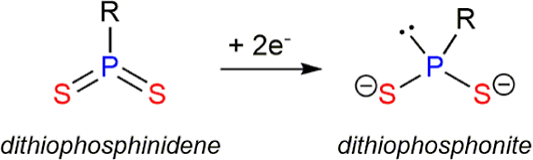
Dithiophosphinidene vs Dithiophosphonite Ligands.

Concerning PRS_2_ ligands stemming from phosphinidene precursors, it is not an obvious matter whether they should be formulated as neutral dithiophosphinidene groups or rather as dithiophosphonite anions, the latter having two extra electrons that could arise from the internal electron transfer from the metal atoms (Chart 3). In the case of **4**, we favor the first formulation based on the fact that the P1–S1 distance of 2.019(2) Å approaches the distances found for conventional P=S bonds (ca. 1.94 Å for compounds **6**, see below). At the same time, although the P1–S2 distance of 2.093(2) Å approaches the reference value of 2.12 Å for a P–S single bond,^22^ this can be interpreted as the result of a strong η^2^ binding of the second P=S bond to the molybdenum atom. Moreover, the degree of pyramidalization of the P atom in **4** is modest [Σ(X–P–S) = 338.6°, with X = C, S]. For comparison, such degree in the dithiophosphonite isomer **5** is noticeably larger [Σ(X–P–S) = 293.6°, see below].

Spectroscopic data in solution for compound **4** (Table 1 and Experimental Section) are consistent with the solid-state structure discussed above and comparable to those of thiophosphinidene complex **3a**, deserving no detailed comments. We just note the moderate (expected) increase of ca. 8 cm^–1^ in the average C–O stretching frequency of the carbonyl ligands and the significant deshielding (ca. 70 ppm) of the ^31^P nucleus when going from **3a** to **4**, the latter being perhaps related to the less strained geometry around the P atom in the dithiophosphinidene complex.

### Structure of Dithiophosphonite Complex **5**

**Figure 4:**
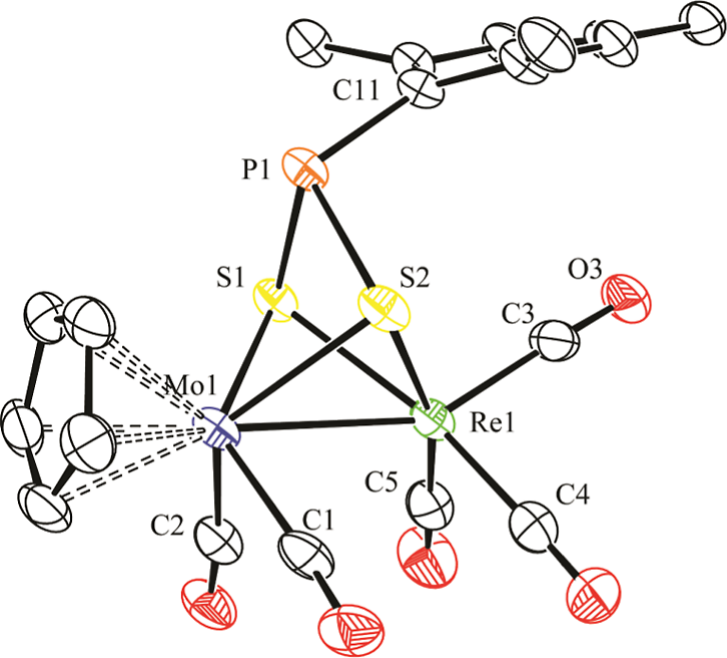
ORTEP diagram (30% probability) of compound **5**, with ^*t*^Bu groups (except their C^1^ atoms) and H atoms omitted for clarity.

The molecule of this complex in the crystal (Figure 4 and Table 5) can be derived from that of its isomer **4** by displacement of the Mo-bound P atom by the S atom terminally bound to Re previously, which now becomes bridging and equivalent to the other (bridging) S atom. The uncoordinated P atom displays an environment strongly pyramidalized, as noted above (Σ(X–P–S) = 293.6°), and can be guessed as bearing a lone electron pair, while the bulky aryl ring points away from the Cp ligand to minimize steric repulsions (*anti* conformation). These geometrical features indicate that the S_2_PR ligand should be more properly described here as a dithiophosphonite ligand, bound to the dimetal center in the novel μ-κ^2^_S,S′_:κ^2^_S,S′_ coordination mode (**D** in Chart 2), much in the same way as two bridging thiolate ligands would do. Accordingly, the P–S distances of ca. 2.17 Å are significantly longer than those in **3a** or **4**, as they now correspond to conventional single bonds (Chart 3), they being actually above the reference value of 2.12 Å for P–S single bonds.^22^ In all, the structure of **5** is comparable to that of the phosphanide- and thiolate-bridged complex [MoReCp(μ-PCy_2_)(μ-SPh) (CO)_5_] mentioned previously,^23^ although the intermetallic distance in **5** is significantly shorter [2.8655(7) vs 2.9702(8) Å]. This shortening is likely the result of the smaller steric requirements of the dithiophosphonite ligand (vs PCy_2_ + SPh) and of the slightly lower covalent radius of S (vs P). Actually, the intermetallic length in **5** appears to be the shortest one measured so far for a Mo–Re single bond in a carbonyl complex (but see below).^29,30^ The structure of **5** can also be related to the structure of the triiron complex [Fe_3_(μ_3_-S_2_PR) (CO)_10_], a molecule made from Lawesson′s reagent (P_2_S_4_R_2_; R = *p*-C_6_H_4_OMe) and [Fe_2_(CO)_9_] and displaying a dithiophosphonite ligand bridging *three* metal atoms in the μ_3_-κ^2^_S,S′_:κ^2^_S,S′_:κ1_P_ coordination mode.^31^ We finally note that despite the different electron counts of the MoCp(CO)_2_ and Re(CO)_3_ fragments (15 and 13 electrons, respectively), the Mo–S lengths in **5** (ca. 2.50 Å) can be considered identical to the Re–S ones (ca. 2.47 Å) after allowing for the ca. 0.03 Å difference in the covalent radii of the metal atoms.^22^ This electronic mismatch seems to be balanced by a stronger coordination of the carbonyl ligands to the Re atom (ca. 1.93 vs 2.00 Å), and perhaps also by the intermetallic bond, likely to be of a significant dative nature.

**Table 5:** Selected Bond Lengths (Å) and Angles (°) for Compound **5**

Mo1–Re1	2.8655(7)	Mo1–S1–Re1	70.56(5)
Mo1–S1	2.493(2)	Mo1–S2–Re1	70.43(5)
Mo1–S2	2.504(2)	S1–Mo1–C1	130.4(2)
Mo1–C1	2.010(9)	S1–Mo1–C2	83.6(2)
Mo1–C2	2.009(9)	S1–Re1–C3	103.7(2)
Re1–S1	2.468(2)	S1–Re1–C4	163.7(3)
Re1–S2	2.465(2)	S1–Re1–C5	98.7(3)
Re1–C3	1.929(9)	C1–Mo1–C2	82.2(4)
Re1–C4	1.928(10)	C3–Re1–C4	90.4(3)
Re1–C5	1.921(11)	C3–Re1–C5	89.1(4)
P1–S1	2.186(2)	S1–P1–S2	83.21(9)
P1–S2	2.165(3)	S1–P1–C11	103.4(2)
P1–C11	1.866(7)	S2–P1–C11	106.9(2)

The IR and NMR data in solution for **5** are consistent with the solid-state structure discussed above and are generally similar to those of compounds **3** and **4**. However, the NMR spectra now indicates the presence of a mirror plane in the molecule relating pairs of atoms in the Mo-bound carbonyls, the aryl ring, and the *ortho*-^*t*^Bu groups of the bridging ligand, and the average C–O stretching frequency of the carbonyl ligands is a bit higher (ca. 5 cm^–1^) as expected. There are, however, two salient spectroscopic features. First, a very large one-bond P–C coupling of 100 Hz for the dithiophosphonite ligand, comparable to the value measured for **2**, which can be analogously related to the presence of a lone pair on the P atom. Second, an unexpectedly strong deshielding of the ^31^P NMR resonance, which now appears at 336.2 ppm, some 295 ppm above that of its dithiophosphinidene isomer **4**. To exclude the possibility that the structure of **5** in solution could be significantly different from the solid-state one, we performed its optimization using DFT methods (see the Experimental Section and the Supporting Information), and found a structure very similar to the experimental one. Interestingly, this structure has a Gibbs free energy at 295 K only 7 kJ/mol below that of the optimized structure of **4**, in spite of the significant differences in the coordination of the PRS_2_ ligands in both molecules. Moreover, the computed chemical shifts reproduced satisfactorily the observed relations (Table 6), with the computed shift for **5** being some 260 ppm above that of **4**. As it can be appreciated from Table 6, the large difference between the magnetic shieldings (σ_calc_) of the ^31^P nuclei in isomers **4** and **5** does not arise from the diamagnetic contributions to the shielding (σ^d^), which are similar to each other but from the paramagnetic ones (σ^p^), 270 ppm more negative for **5**. This could have been hardly anticipated on simple chemical grounds, such as the appearance of a lone pair at the P atom when going from **4** to **5**. Indeed, previous work from us on diphosphorus- and triphosphorus-bridged complexes has revealed that the chemical shifts of these lone-pair-bearing P atoms are largely unpredictable because of the dramatic variations in the paramagnetic contributions to their magnetic nuclear shielding.^32^

**Table 6:** DFT-Computed ^31^P NMR Parameters for Compounds **4** to **6**^a^

compound	σ^d^	σ^p^	σ_calc_	δ_calc_	Δδ
**4**	981.1	–701.5	279.7	41.4	0
**5**	988.8	–970.3	18.5	302.5	33.7
***anti*-6a**	984.4	–833.4	150.9	170.1	20.4

a δ_calc_ = σ_ref_ – σ_calc_; Δδ = δ_exp_ – δ_calc_. The experimental chemical shift of 41.4 ppm of compound **4** relative to H_3_PO_4_ was used to calibrate the magnetic shielding of the reference (σ_ref_ = 321.1 ppm).

### Structure of Trithiophosphonate Complexes ***syn***- and ***anti*-6**

**Figure 5:**
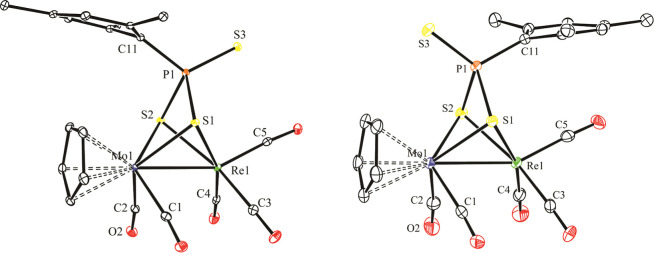
ORTEP diagram (30% probability) of compounds ***syn*-6** (left) and ***anti*-6** (right), with ^*t*^Bu groups (except their C^1^ atoms) and H atoms omitted for clarity.

**Chart 4:**
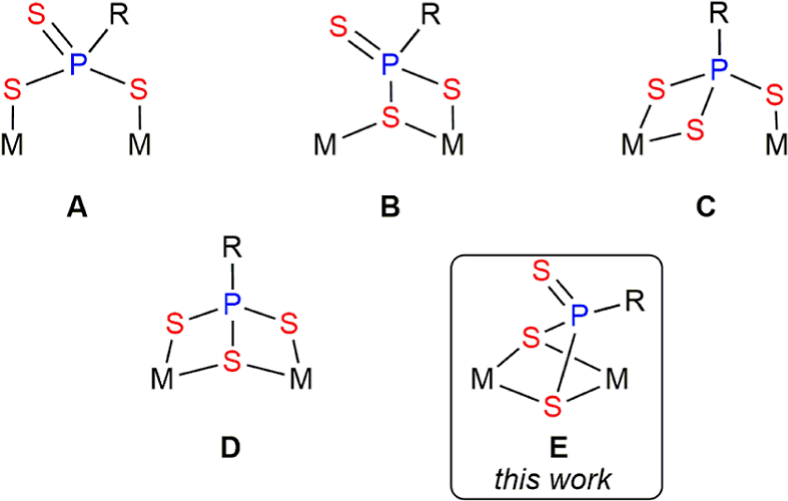
Coordination modes of bridging PRS_3_ ligands.

The molecule of the *anti* isomer of **6** can be just derived by adding a sulfur atom to the P atom of **5**, while that of the *syn* isomer is generated by just exchanging the terminal S and the aryl group around phosphorus in the *anti* isomer (Figure 5 and Table 7). Interatomic distances in these isomers are very similar to each other as expected and are also comparable to those measured in the dithiophosphonite precursor **5**, with Mo–S distances of ca. 2.51 Å, Re–S distances of ca. 2.48 Å (isomer *anti*) and 2.50 Å (isomer *syn*), and distances of P to the bridging S atoms of ca. 2.15 Å, very close to the reference value of ca. 2.12 Å for P–S single bonds. In contrast, the S atoms terminally bound to phosphorus in both isomers of **6** display much shorter P–S lengths of ca. 1.93 Å, as expected for P=S double bonds. For comparison, the P=S length in the digold complex [Au_2_(μ-κ^1^_S_:κ^1^_S′_-S_3_PPh)(PPh_3_)_2_] is 1.932(2) Å.^33^ We note that in the latter complex, the trithiophosphonate ligand acts as a two-electron donor (**A** in Chart 4). Other crystallographically characterized trithiophosphonate-bridged complexes can be classified as displaying four-electron donor ligands of type μ-κ^2^_S,S′_:κ^1^_S_ (**B**),^34^ or μ-κ^2^_S,S′_:κ^1^_S″_ (**C**)^35^ and six-electron donor ligands of type μ-κ^2^_S,S′_:κ^2^_S,S″_ (**D**).^36^ The coordination mode of the six-electron donor trithiophosphonate ligand in complexes **6** is different from the above ones, and it appears to have been not identified previously, therefore adding to the set of possible coordination modes of these versatile bridging ligands (**E** in Chart 4). We finally note that the intermetallic lengths of ca. 2.84 Å in isomers **6** are a bit shorter than the corresponding one in precursor **5**, thus setting a new lower limit for Mo–Re single bond lengths in binuclear carbonyl complexes.

**Table 7:** Selected Bond Lengths (Å) and Angles (°) for Compounds ***syn***- and *anti*-**6**

	*syn*-6	*anti*-6		*syn*-6	*anti*-6
Mo1–Re1	2.8369(2)	2.8443(3)	Mo1–S1–Re1	68.99(1)	69.69(2)
Mo1–S1	2.5010(5)	2.5038(8)	Mo1–S2–Re1	69.16(1)	69.31(2)
Mo1–S2	2.5090(5)	2.5147(8)	S1–Mo1–C1	82.06(6)	78.6(1)
Mo1–C1	2.001(2)	1.987(4)	S1–Mo1–C2	129.66(6)	128.7(1)
Mo1–C2	2.012(2)	2.008(4)	S1–Re1–C3	100.18(6)	97.5(1)
Re1–S1	2.5083(5)	2.4739(7)	S1–Re1–C4	163.01(6)	165.7(1)
Re1–S2	2.4892(5)	2.4870(8)	S1–Re1–C5	104.49(6)	104.5(1)
Re1–C3	1.920(2)	1.908(4)	C1–Mo1–C2	82.22(8)	82.7(2)
Re1–C4	1.910(2)	1.919(4)	C3–Re1–C4	89.99(9)	88.7(2)
Re1–C5	1.917(2)	1.921(4)	C3–Re1–C5	87.82(9)	88.6(2)
P1–S1	2.1451(7)	2.144(1)	S1–P1–S2	86.16(3)	85.57(4)
P1–S2	2.1439(7)	2.157(1)	S3–P1–S1	113.85(3)	114.01(5)
P1–S3	1.9333(7)	1.935(1)	S3–P1–S2	117.32(3)	117.67(5)
P1–C11	1.847(2)	1.844(3)	S3–P1–C11	117.86(7)	117.0(1)

Spectroscopic data in solution for the *syn* and *anti* isomers of **6** are consistent with the solid-state structures discussed above, and comparable to each other, while just revealing two remarkable differences with respect to precursor **5**. First, their C–O stretches are some 7 cm^–1^ higher, which is likely a consequence of the formal increase in the oxidation state of the P atom (from +3 to +5) when going from **5** to **6**. Second, the P nuclei in isomers **6** are substantially more shielded (by ca. 150 ppm) than in compound **5**, a difference supported by DFT calculations (Table 6). Such a shielding effect is qualitatively similar to the ones commonly observed for phosphites upon conversion into the corresponding oxides or sulfides but opposite to the deshielding effect usually observed for phosphines upon oxidation.^37^ We finally note the substantial deshielding of the cyclopentadienyl protons of ***anti*-6** compared to those of its *syn* isomer (δ_H_ 6.20 and 5.31 ppm, respectively, in CD_2_Cl_2_ solution). This difference is likely caused by the positioning of the corresponding H atoms in the *anti* isomer close to the uncoordinated S atom of the trithiophosphonate ligand.

### Reactions of Compounds **1** with Selenium

**Scheme 7:**
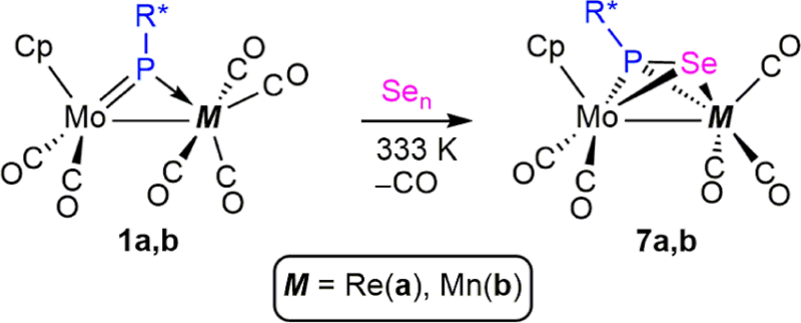
Selenophosphinidene Derivatives of Compounds **1**

Reactions of compounds **1a,b** with a moderate excess of gray selenium proceeded smoothly at 333 K in toluene solution (faster for the Mn complex) to give the corresponding selenophosphinidene-bridged complexes [MoMCp(μ-η^2^:η^2^-SePR*)(CO)_5_] as unique products [M = Re (**7a**), Mn (**7b**)], which were isolated as crystalline solids in ca. 70% yield upon crystallization (Scheme 7). No evidence for the incorporation of more than one Se atom to the parent complexes was obtained when using either larger amounts of selenium or higher temperatures (boiling toluene solutions).

**Figure 6:**
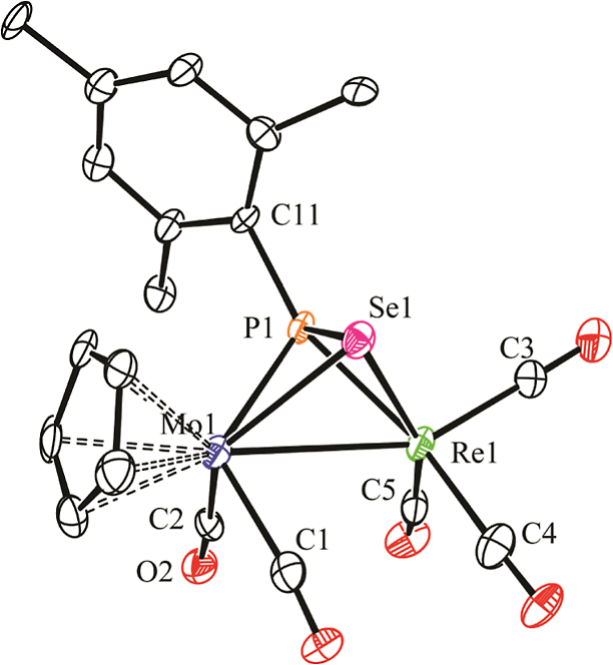
ORTEP diagram (30% probability) of compound **7a**, with ^*t*^Bu groups (except their C^1^ atoms) and H atoms omitted for clarity.

The molecule of rhenium complex **7a** in the crystal (Figure 6 and Table 8) is fully comparable to that of the thiophosphinidene complex **3b** if we just replace Mn with Re and S with Se atoms in the latter and allow for the differences in the covalent radii of these pairs of atoms. We should note, in addition, that this complex provides the first crystallographic characterization of a selenophosphinidene ligand in the μ-η^2^:η^2^ coordination mode. As a result of this coordination, the PR*Se ligand formally provides the dimetal site with six electrons (two lone pairs from P and Se atoms and the π-bonding electrons of the P=Se double bond), which then becomes electron-precise (34 electrons) in agreement with the intermetallic length of 3.109(2) Å, consistent with the formulation of a Mo–Re single bond.^29^ Besides this, we note that the P–Se separation of 2.241(5) Å in **7a** is substantially longer than the P=Se distance in complex [FeCp{κ^1^_P_–P(Se)(OR)_2_}(CO)_2_] (2.117(2) Å)^38^ and instead approaches the reference value of ca. 2.27 Å for a single bond between these atoms.^22^ This suggests that the interaction of the π-bonding electrons of the selenophosphinidene ligand with the dimetal center is very strong, seemingly stronger than the η^2^-interaction of the selenophosphinidene ligand in complex [Mo_2_Cp_2_(μ-κ^1^_P_:η^2^-SePH)(CO)_2_(η^6^-R*H)] (P–Se = 2.199(2) Å).^12a^

**Table 8:** Selected Bond Lengths (Å) and Angles (°) for Compound **7a**

Mo1–Re1	3.109(2)	Mo1–P1–Re1	79.88(16)
Mo1–P1	2.475(6)	Mo1–Se1–Re1	72.79(6)
Mo1–Se1	2.608(2)	P1–Mo1–C1	120.1(6)
Mo1–C1	2.00(2)	P1–Mo1–C2	88.5(6)
Mo1–C2	1.98(2)	P1–Re1–C3	104.2(7)
Re1–P1	2.367(4)	P1–Re1–C4	153.0(7)
Re1–Se1	2.633(2)	P1–Re1–C5	107.5(6)
Re1–C3	1.92(3)	C1–Mo1–C2	81.9(8)
Re1–C4	1.96(3)	C3–Re1–C4	92.9(12)
Re1–C5	1.91(2)	C3–Re1–C5	90.3(10)
P1–Se1	2.241(5)		
P1–C11	1.82(2)		

Spectroscopic data in solution for compounds **7a** and **7b** are similar to each other, essentially consistent with the solid-state structure of **7a** just discussed and comparable to those of the related thiophosphinidene complexes **3a,b**, deserving no detailed analysis. We just note that the ^31^P chemical shifts of complexes **7** are ca. 50 ppm higher than the shifts in the related thiophosphinidene complexes **3**, a difference substantially larger than the ones observed in different κ^1^_P_:η^2^-bridged selenophosphinidene Mo_2_ complexes (Δδ in the range 8–5 ppm).^12a^ In contrast, the one-bond P–Se couplings in compounds **7** (ca. 310 Hz) are substantially lower than the values recorded for the mentioned Mo_2_ complexes (390–430 Hz). The latter is consistent with the increase in the coordination number of Se (from 2 to 3) when going from the μ-κ^1^_P_:η^2^- to μ-η^2^:η^2^-coordination mode of the selenophosphinidene ligand^20^ and with the longer P–Se distance in the latter case.

## Concluding Remarks

The stepwise sulfurization of the phosphinidene ligand in complexes [MoMCp(μ-PR*) (CO)_6_] (M = Re, Mn) is likely initiated in all cases by insertion of sulfur into the M–P bond of the parent complex to yield hexacarbonyl complexes of type [MoMCp(μ-η^2^:κ^1^_S_-SPR*)(CO)_6_], only detected when M = Re, containing a thiophosphinidene ligand unexpectedly bridging the dimetal center in the novel μ-κ^1^_S_:η^2^ coordination mode, as opposed to the μ-κ^1^_P_:η^2^ mode usually found in previous PRS-bridged complexes. The latter are decarbonylated easily to give the pentacarbonyl derivatives [MoMCp(μ-η^2^:η^2^-SPR*)(CO)_5_], whereby the thiophosphinidene ligand rearranges into the rare six-electron donor μ-η^2^:η^2^ coordination mode, thus keeping constant the electron count of the binuclear complex. Related reactions take place when using selenium to give analogous selenophosphinidene-bridged pentacarbonyl complexes [MoMCp(μ-η^2^:η^2^-SePR*)(CO)_5_], even when using excess selenium. However, the use of excess sulfur in the reactions of the MoRe complex leads to the incorporation of two or three S atoms depending on reaction conditions, an unusual behavior for phosphinidene-bridged homometallic complexes of any type. The influence of the different experimental variables on the final output of these reactions suggests that the thiophosphinidene ligand in the pentacarbonyl complex [MoReCp(μ-η^2^:η^2^-SPR*)(CO)_5_] is further sulfurized at least through two competing reaction paths of different rates. The slower one would involve insertion of a second S atom into the Re–P bond to give the dithiophosphinidene complex [MoReCp(μ-η^2^:κ^2^_S,S′_-S_2_PR*)(CO)_5_], followed by rearrangement of the latter into the slightly more stable dithiophosphonite isomer [MoReCp(μ-κ^2^_S,S′_:κ^2^_S,S′_-S_2_PR*)(CO)_5_], which in turn would add a third S atom at its lone-pair bearing P atom to yield the trithiophosphonate derivative [MoReCp(μ-κ^2^_S,S′_:κ^2^_S,S′_-S_3_PR*)(CO)_5_]. In contrast, the faster reaction path would connect the thiophosphinidene pentacarbonyl complex with the trithiophosphonate derivative without detectable intermediate species, but we cannot offer a reliable hypothesis about the elemental steps involved in this faster transformation.

## Experimental Section

### General Procedures and Starting Materials

General experimental procedures as well as the preparation of compounds [MoReCp(μ-PR*)(CO)_6_] (**1a**) and [MoMnCp(μ-PR*)(CO)_6_] (**1b**) were carried out as described previously (Cp = η^5^-C_5_H_5_; R* = 2,4,6-C_6_H_2_^*t*^Bu_3_).^5,7^

#### Preparation of [MoReCp(μ-η^2^:κ^1^_S_-SPR*)(CO)_5_] (**2**)

*Method A*: elemental sulfur (0.0016 g, 0.0062 mmol of S_8_) and compound **1a** (0.040 g, 0.051 mmol) were dissolved in toluene (8 mL) in a Schlenk tube equipped with a Young′s valve. After closing the valve, the mixture was stirred at 313 K for 15 days to give an orange solution. The solvent was then removed under vacuum, the residue was extracted with dichloromethane/petroleum ether (1/8), and the extracts were chromatographed on alumina at 258 K. Elution with the same solvent mixture gave first a brown fraction containing a significant amount of unreacted **1a**, then an orange fraction yielding, after removal of solvents, compound **2** as an orange solid (0.015 g, 36%). This product was invariably contaminated with a small amount of an unidentified product, so no elemental analysis of it was recorded. *Method B*: A toluene solution (5 mL) of compound **3a** (0.020 g, 0.025 mmol) was placed in a Schlenk tube equipped with a Young′s valve. After freezing the solution, the inert atmosphere was removed under vacuum and replaced with CO. Then the valve was closed and the tube was allowed to reach room temperature. The solution was then stirred at room temperature for 3 h to give an orange solution yielding, after filtration and removal of the solvent, an orange residue containing an almost pure compound **2** (0.017 g, 83%). The crystals used in the X-ray study of **2** were grown through diffusion of a layer of petroleum ether into a concentrated toluene solution of the complex at 253 K. Anal. Calcd for C_29_H_34_MoO_6_PReS: C, 42.28; H, 4.16; S, 3.89. Found: C, 42.03; H, 3.95; S, 3.71. ^1^H NMR (400.13 MHz, CD_2_Cl_2_): δ 7.18, 7.06 (2s, vbr, 2H, C_6_H_2_), 4.90 (s, 5H, Cp), 1.64, (s, br, 18H, *o*-^*t*^Bu), 1.25 (s, 9H, *p*-^*t*^Bu). ^1^H NMR (400.13 MHz, CD_2_Cl_2_, 243 K): δ 7.21, 7.02 (2s, 2 × 1H, C_6_H_2_), 4.92 (s, 5H, Cp), 1.72, 1.57, 1.25 (3s, 3 × 9H, ^*t*^Bu). ^13^C{^1^H} NMR (100.63 MHz, CD_2_Cl_2_, 243 K): δ 239.0 (d, *J*_CP_ = 14, MoCO), 229.3 (s, MoCO), 188.1 (s, ReCO), 185.7 (d, *J*_CP_ = 24, ReCO), 185.7 (d, *J*_CP_ = 6, ReCO), 184.8 (s, ReCO), 156.9 [s, C^2^(C_6_H_2_)], 148.7 [d, *J*_CP_ = 97, C^1^(C_6_H_2_)], 148.6 [s, C^4^(C_6_H_2_)], 122.9 [d, *J*_CP_ = 12, C^3^(C_6_H_2_)], 93.7 (s, Cp), 35.7 [s, br, C^1^(*o*-^*t*^Bu)], 34.8 [s, C^1^(*p*-^*t*^Bu)], 33.7 [s, br, C^2^(*o*-^*t*^Bu)], 31.1 [s, C^2^(*p*-^*t*^Bu)]. ^13^C{^1^H} NMR (100.63 MHz, CD_2_Cl_2_, 243 K): δ 239.5 (d, *J*_CP_ = 12, MoCO), 229.6 (s, MoCO), 188.4 (s, ReCO), 185.7 (d, *J*_CP_ = 24, ReCO), 185.5 (s, br, ReCO), 185.0 (s, ReCO), 156.7 [d, *J*_CP_ = 8, C^2,6^(C_6_H_2_)], 156.1 [s, C^6,2^(C_6_H_2_)], 148.6 [d, *J*_CP_ = 96, C^1^(C_6_H_2_)], 148.2 [s, C^4^(C_6_H_2_)], 123.8 [s, C^3,5^(C_6_H_2_)], 120.8 [s, C^5,3^(C_6_H_2_)], 93.7 (s, Cp), 40.5, 39.4 [2s, C^1^(^*t*^Bu)], 35.8 [s, br, C^2^(^*t*^Bu)], 34.7 [s, C^1^(^*t*^Bu)], 33.3 [d, *J*_CP_ = 12, C^2^(^*t*^Bu)], 30.9 [s, C^2^(^*t*^Bu)].

#### Preparation of [MoReCp(μ-η^2^:η^2^-SPR*)(CO)_5_] (**3a**)

Elemental sulfur (0.0012 g, 0.0047 mmol of S_8_) and compound **1a** (0.030 g, 0.038 mmol) were dissolved in toluene (8 mL), and the mixture was stirred at 363 K for 1 h to give a brown-orange solution which was filtered. The solvent was then removed under vacuum, and the residue was crystallized by the slow diffusion of a layer of petroleum ether into a concentrated toluene solution of this crude product, which yielded compound **3a** as an orange crystalline solid (0.017 g, 56%). Anal. Calcd for C_28_H_34_MoO_5_PReS: C, 42.26; H, 4.31; S, 4.03. Found: C, 41.95; H, 3.98; S, 3.92. ^1^H NMR (400.13 MHz, CD_2_Cl_2_): δ 7.32, 7.31 (2s, 2 × 1H, C_6_H_2_), 5.00 (s, 5H, Cp), 1.60, 1.54, 1.30 (3s, 3 × 9H, ^*t*^Bu). ^13^C{^1^H} NMR (100.63 MHz, CD_2_Cl_2_): δ 230.0 (d, *J*_CP_ = 5, MoCO), 228.0 (s, MoCO), 197.2 (s, br, 3ReCO), 160.1 [d, *J*_CP_ = 4, C^2,6^(C_6_H_2_)], 159.5 [d, *J*_CP_ = 15, C^4^(C_6_H_2_)], 151.8 [d, *J*_CP_ = 4, C^6,2^(C_6_H_2_)], 124.4 [d, *J*_CP_ = 7, C^3,5^(C_6_H_2_)], 123.0 [d, *J*_CP_ = 13, C^5,3^(C_6_H_2_)], 113.3 [d, *J*_CP_ = 36, C^1^(C_6_H_2_)], 91.7 (s, Cp), 40.3, 40.1, 35.1 [3s, C^1^(^*t*^Bu)], 34.4 [s, C^2^(^*t*^Bu)], 33.6 [d, *J*_CP_ = 7, C^2^(^*t*^Bu)], 31.0 [s, C^2^(^*t*^Bu)].

#### Preparation of [MoMnCp(μ-η^2^:η^2^-SPR*)(CO)_5_] (**3b**)

Elemental sulfur (0.008 g, 0.031 mmol of S_8_) and compound **1b** (0.020 g, 0.030 mmol) were dissolved in toluene (8 mL), and the mixture was stirred at 333 K for 45 min to give an orange solution. Workup as described for **3a** gave compound **3b** as orange X-ray quality crystals (0.015 g, 75%). Anal. Calcd for C_28_H_34_MoMnO_5_PS: C, 50.61; H, 5.16; S, 4.83. Found: C, 50.31; H, 4.54; S, 5.04. ^1^H NMR (300.13 MHz, CD_2_Cl_2_, 233 K): δ 7.34 (d, *J*_HP_ = 7, 1H, C_6_H_2_), 7.29 (s, 1H, C_6_H_2_), 4.97 (s, 5H, Cp), 1.61, 1.53, 1.30 (3s, 3 × 9H, ^*t*^Bu). ^13^C{^1^H} NMR (100.63 MHz, CD_2_Cl_2_, 233 K): δ 233.4 (d, *J*_CP_ = 7, MoCO), 230.0 (s, MoCO), 223.1 (s, br, 3MnCO), 159.6 [d, *J*_CP_ = 16, C^4^(C_6_H_2_)], 158.8 [s, br, C^2,6^(C_6_H_2_)], 151.2 [d, *J*_CP_ = 4, C^6,2^(C_6_H_2_)], 124.1 [s, C^3,5^(C_6_H_2_)], 123.2 [d, *J*_CP_ = 13, C^5,3^(C_6_H_2_)], 119.3 [d, *J*_CP_ = 35, C^1^(C_6_H_2_)], 91.6 (s, Cp), 39.6 [s, 2C^1^(^*t*^Bu)], 35.0 [s, C^1^(^*t*^Bu)], 34.1 [s, C^2^(^*t*^Bu)], 33.2 [d, *J*_CP_ = 6, C^2^(^*t*^Bu)], 30.8 [s, C^2^(^*t*^Bu)].

#### Preparation of [MoReCp(μ-η^2^:κ^2^_S,S′_-S_2_PR*)(CO)_5_] (**4**)

Elemental sulfur (0.016 g, 0.062 mmol of S_8_) and compound **1a** (0.050 g, 0.063 mmol) were dissolved in toluene (8 mL), and the mixture was stirred at 333 K for 2 h to give a brown solution. After removal of the solvent under vacuum, the residue was extracted with dichloromethane/petroleum ether (1/10), and the extracts were chromatographed on alumina at 258 K. Elution with the same solvent mixture gave first a yellow fraction containing small amounts of [MoReCp(μ-H){μ-P(CH_2_CMe_2_)C_6_H_2_^*t*^Bu_2_}(CO)_6_]^5^ and then minor orange and red fractions containing unidentified species. Elution with dichloromethane/petroleum ether (1/5) gave two separated orange fractions. Removal of solvents from the latter fractions yielded compounds **3a** (0.013 g, 26%) and **4** (0.020 g, 38%), both as orange microcrystalline solids. X-ray quality crystals of **4** were grown as described for **2** (from dichloromethane/petroleum ether). Anal. Calcd for C_28_H_34_MoO_5_PReS_2_: C, 40.62; H, 4.14; S, 7.75. Found: C, 40.89; H, 4.39; S, 8.15. ^1^H NMR (400.13 MHz, CD_2_Cl_2_): δ 7.37 (s, 1H, C_6_H_2_), 7.23 (dd, *J*_HP_ = 6, *J*_HH_ = 2, 1H, C_6_H_2_), 4.97 (s, 5H, Cp), 1.73, 1.57, 1.29 (3s, 3 × 9H, ^*t*^Bu). ^13^C{^1^H} NMR (100.63 MHz, CD_2_Cl_2_): δ 237.8 (d, *J*_CP_ = 18, MoCO), 226.0 (d, *J*_CP_ = 6, MoCO), 193.0 (s, br, 3ReCO), 157.6 [d, *J*_CP_ = 18, C^1^(C_6_H_2_)], 157.3 [s, C^2,6^(C_6_H_2_)], 153.2 [s, C^4^(C_6_H_2_)], 149.5 [d, *J*_CP_ = 19, C^6,2^(C_6_H_2_)], 125.5 [d, *J*_CP_ = 10, C^3,5^(C_6_H_2_)], 121.4 [d, *J*_CP_ = 14, C^5,3^(C_6_H_2_)], 93.1 (s, Cp), 42.8, 40.3, 35.4 [3s, C^1^(^*t*^Bu)], 34.3, 34.2, 30.9 [3s, C^2^(^*t*^Bu)].

#### Preparation of [MoReCp(μ-κ^2^_S,S′_:κ^2^_S,S′_-S_2_PR*)(CO)_5_] (**5**)

Elemental sulfur (0.005 g, 0.019 mmol of S_8_) and compound **1a** (0.030 g, 0.038 mmol) were dissolved in toluene (8 mL), and the mixture was refluxed for 20 min to give a brown solution. After removal of the solvent under vacuum, the residue was extracted with dichloromethane/petroleum ether (1/4), and the extracts were chromatographed on alumina at 258 K. Elution with the same solvent mixture gave first a yellow fraction and then an orange fraction. Removal of solvents from the latter fractions yielded compounds ***anti*-6** (0.008 g, 24%) and **5** (0.010 g, 32%) as yellow and orange microcrystalline solids, respectively. X-ray quality crystals of **5** were grown as described for **2** (from dichloromethane/diethyl ether/petroleum ether). Anal. Calcd for C_28_H_34_MoO_5_PReS_2_: C, 40.62; H, 4.14; S, 7.75. Found: C, 40.35; H, 4.01; S, 7.47. ^1^H NMR (300.13 MHz, CD_2_Cl_2_): δ 7.30 (d, *J*_HP_ = 2, 2H, C_6_H_2_), 6.13 (s, 5H, Cp), 1.36 (s, br, 18H, *o*-^*t*^Bu), 1.34 (s, 9H, *p*-^*t*^Bu). ^13^C{^1^H} NMR (100.63 MHz, CD_2_Cl_2_): δ 232.5 (d, *J*_CP_ = 4, MoCO), 196.2 (s, br, 3ReCO), 152.3 [d, *J*_CP_ = 6, C^2^(C_6_H_2_)], 151.2 [s, C^4^(C_6_H_2_)], 149.5 [d, *J*_CP_ = 100, C^1^(C_6_H_2_)], 124.9 [s, C^3^(C_6_H_2_)], 93.9 (s, Cp), 39.8 [d, *J*_CP_ = 4, C^1^(*o*-^*t*^Bu)], 34.8 [s, C^1^(*p*-^*t*^Bu)], 32.9 [d, *J*_CP_ = 10, C^2^(*o*-^*t*^Bu)], 31.3 [s, C^2^(*p*-^*t*^Bu)].

#### Preparation of *anti*-[MoReCp(μ-κ^2^_S,S′_:κ^2^_S,S′_-S_3_PR*)(CO)_5_] (***anti*-6**)

Elemental sulfur (0.005 g, 0.019 mmol of S_8_) and compound **1a** (0.040 g, 0.051 mmol) were dissolved in toluene (8 mL), and the mixture was stirred at 363 K for 16 h to give a brown solution. After removal of the solvent under vacuum, the residue was extracted with dichloromethane/petroleum ether (1/6), and the extracts were chromatographed on alumina at 258 K. Elution with the same solvent mixture gave first minor yellow and black fractions containing unidentified species and then a major yellow fraction. Removal of solvents from the latter fraction yielded compound ***anti*-6** (0.020 g, 46%) as a yellow microcrystalline solid. X-ray quality crystals of ***anti*-6** were grown as described for **4**. Anal. Calcd for C_28_H_34_MoO_5_PReS_3_: C, 39.11; H, 3.99; S, 11.19. Found: C, 39.23; H, 3.54; S, 10.93. ^1^H RMN (CD_2_Cl_2_, 300.09 MHz): δ 7.45 (d, *J*_HP_ = 6, 2H, C_6_H_2_), 6.20 (s, 5H, Cp), 1.65 (s, 18H, *o*-^*t*^Bu), 1.28 (s, 9H, *p*-^*t*^Bu). ^1^H NMR (400.13 MHz, toluene-*d*_8_): δ 7.54 (d, *J*_HP_ = 6, 2H, C_6_H_2_), 5.21 (s, 5H, Cp), 1.75 (s, 18H, *o*-^*t*^Bu), 1.30 (s, 9H, *p*-^*t*^Bu). ^13^C{^1^H} NMR (100.63 MHz, toluene-*d*_8_): δ 230.4 (s, MoCO), 194.1 (s, br, 3ReCO), 153.2 [s, C^4^(C_6_H_2_)], 152.2 [d, *J*_CP_ = 10, C^2^(C_6_H_2_)], 144.9 [d, *J*_CP_ = 45, C^1^(C_6_H_2_)], 95.3 (s, Cp), 42.0 [s, C^1^(*o*-^*t*^Bu)], 36.5 [s, C^2^(*o*-^*t*^Bu)], 35.2 [s, C^1^(*p*-^*t*^Bu)], 31.3 [s, C^2^(*p*-^*t*^Bu)]. Resonances for the C^3^(C_6_H_2_) carbons could not be clearly identified in the spectrum, as they were obscured by those of the ring carbons of the solvent.

#### Preparation of *syn*-[MoReCp(μ-κ^2^_S,S′_:κ^2^_S,S′_-S_3_PR*)(CO)_5_] (***syn*-6**)

Compound ***anti*-6** (0.020 g, 0.023 mmol) was dissolved in dichloromethane to reach, after 12 h at room temperature, an equimolar equilibrium mixture of *syn* and *anti* isomers. After removal of the solvent, the residue was washed with dichloromethane/petroleum ether (1/8) to yield isomer ***syn*-6** as a yellow solid (0.008 g, 40%). The crystals used in the X-ray study of ***syn*-6** were grown through crystallization of a concentrated dichloromethane solution of the complex at 253 K. Anal. Calcd for C_28_H_34_MoO_5_PReS_3_: C, 39.11; H, 3.99; S, 11.19. Found: C, 38.90; H, 3.65; S, 10.87. ^1^H NMR (300.13 MHz, CD_2_Cl_2_): δ 7.60 (d, *J*_HP_ = 5, 2H, C_6_H_2_), 5.31 (s, 5H, Cp), 1.65 (s, 18H, *o*-^*t*^Bu), 1.39 (s, 9H, *p*-^*t*^Bu).

#### Preparation of [MoReCp(μ-η^2^:η^2^-SePR*)(CO)_5_] (**7a**)

Gray selenium (0.006 g, 0.076 mmol) and compound **1a** (0.020 g, 0.025 mmol) were dissolved in toluene (8 mL), and the mixture was stirred at 333 K for 6.5 h to give a brown-orange solution. The solvent was then removed under vacuum, the residue was extracted with dichloromethane (3 mL), and the extract filtered. After removal of the solvent from the filtrate, the residue was crystallized by the slow diffusion of a layer of petroleum ether into a concentrated dichloromethane solution of the crude product, yielding compound **7a** as X-ray quality orange crystals (0.014 g, 66%). Anal. Calcd for C_28_H_34_MoO_5_PReSe: C, 39.91; H, 4.07. Found: C, 39.65; H, 3.63. ^1^H NMR (300.13 MHz, CD_2_Cl_2_): δ 7.29 (d, *J*_HP_ = 3, 1H, C_6_H_2_), 7.28 (s, 1H, C_6_H_2_), 5.00 (s, 5H, Cp), 1.59, 1.54 (2s, br, 2 × 9H, *o*-^*t*^Bu), 1.30 (s, 9H, *p*-^*t*^Bu). ^13^C{^1^H} NMR (100.63 MHz, CD_2_Cl_2_): δ 228.4 (d, *J*_CP_ = 6, MoCO), 228.1 (s, MoCO), 196.4 (s, br, 3ReCO), 160.1 [s, C^4^(C_6_H_2_)], 159.1 [d, *J*_CP_ = 15, C^2,6^(C_6_H_2_)], 151.5 [d, *J*_CP_ = 4, C^6,2^(C_6_H_2_)], 124.1 [d, *J*_CP_ = 6, C^3,5^(C_6_H_2_)], 122.9 [d, *J*_CP_ = 13, C^5,3^(C_6_H_2_)], 114.5 [d, *J*_CP_ = 35, C^1^(C_6_H_2_)], 91.0 (s, Cp), 40.3, 40.2 [2s, br, C^1^(*o*-^*t*^Bu)], 35.0 [s, C^1^(*p*-^*t*^Bu)], 34.8 [s, C^2^(*p*-^*t*^Bu)], 33.7 [d, *J*_CP_ = 7, C^2^(*o*-^*t*^Bu)], 31.0 [s, C^2^(*p*-^*t*^Bu)].

#### Preparation of [MoMnCp(μ-η^2^:η^2^-SePR*)(CO)_5_] (**7b**)

Gray selenium (0.007 g, 0.089 mmol) and compound **1b** (0.020 g, 0.030 mmol) were dissolved in toluene (8 mL), and the mixture was stirred at 333 K for 2 h to give a brown-orange solution. Workup as described for **7a** yielded compound **7b** as orange crystals (0.015 g, 70%). Anal. Calcd for C_28_H_34_MoMnO_5_PSe: C, 47.27; H, 4.82. Found: C, 47.07; H, 4.70. ^1^H NMR (300.13 MHz, CD_2_Cl_2_): δ 7.31, 7.27 (AB mult, *J*_HP_ = 6, 2, *J*_HH_ = 2, 2H, C_6_H_2_), 4.93 (s, 5H, Cp), 1.62, 1.53 (2s, br, 2 × 9H, *o*-^*t*^Bu), 1.30 (s, 9H, *p*-^*t*^Bu). ^13^C{^1^H} NMR (100.63 MHz, CD_2_Cl_2_): δ 231.6 (d, *J*_CP_ = 8, MoCO), 230.1 (s, MoCO), 226.0 (s, br, 3MnCO), 159.5 [s, C^2,6^(C_6_H_2_)], 159.4 [s, C^4^(C_6_H_2_)], 151.4 [d, *J*_CP_ = 4, C^6,2^(C_6_H_2_)], 123.9 [d, *J*_CP_ = 6, C^3,5^(C_6_H_2_)], 122.9 [d, *J*_CP_ = 12, C^5,3^(C_6_H_2_)], 121.0 [d, *J*_CP_ = 36, C^1^(C_6_H_2_)], 90.9 (s, Cp), 39.8 [s, 2C^1^(^*t*^Bu)], 35.1 [s, C^1^(^*t*^Bu)], 34.7 [s, C^2^(^*t*^Bu)], 33.6 [d, *J*_CP_ = 6, C^2^(^*t*^Bu)], 30.9 [s, C^2^(^*t*^Bu)].

### X-ray Structure Determination of Compounds **3b**, **4**, **5**, ***anti*-6**, and **7a**

Data collection for these compounds was performed at ca. 150 K on an Oxford Diffraction Xcalibur Nova single-crystal diffractometer, using Cu Kα radiation. Structure solution and refinements were carried out as described before^5,7^ to give the residuals shown in Tables S1 and S2. For compound **3b**, two independent but otherwise similar molecules were present in the unit cell; both of them had a disordered ^*t*^Bu group, satisfactorily modeled over two sites with 0.5/0.5 occupancies. For compound **4**, two ^*t*^Bu groups were also disordered, satisfactorily modeled over two sites with 0.5/0.5 and 0.6/0.4 occupancies, respectively; nevertheless, some restraints had to be applied to the C–C distances to achieve a consistent model. Moreover, a disordered toluene molecule placed on a symmetry element was present, which could not be satisfactorily modeled; therefore, the squeeze procedure,^39^ as implemented in PLATON,^40^ was used. Compound **5** crystallized with two disordered molecules of hexane which could not be satisfactorily modeled; therefore, the squeeze procedure was applied as above. For compound ***anti*-6**, they were four independent but otherwise similar molecules in the unit cell and two dichloromethane molecules; two molecules of the complex had a disordered ^*t*^Bu group each, satisfactorily modeled over two sites with 0.7/0.3 occupancies, although a restraint had to be applied on the C(21B)–C(23B) distance to obtain a consistent model. For compound **7a**, there were two independent but similar molecules in the unit cell, each of them with one disordered ^*t*^Bu group which was satisfactorily modeled over two sites with 0.6/0.4 occupancies, although some restraints had to be applied too on the C–C distances to obtain a consistent model.

### X-ray Structure Determination of Compounds **2** and ***syn*-6**

Data collection for these compounds was performed at 100 K on a Bruker D8 VENTURE Photon III 14 κ-geometry diffractometer using Mo *K*_α_ radiation. The structures were solved using SUPERFLIP,^41^ and refinements were carried out as described before.^5,7^ The unit cell of **2** contains one toluene molecule per unit of complex disordered over two very close positions; such a disorder could not be satisfactorily modeled. There is also a second toluene molecule (half a molecule per unit of complex) located on the edges of the unit cell and disordered over two-symmetry related sites that could be neither modeled properly. Both molecules were then removed from the model by using the squeeze procedure as above. Upon convergence, the strongest residual peak (1.81 eÅ^–3^) was placed around the rhenium atom. Complex ***syn*-6** crystallizes with one molecule of dichloromethane, which was refined satisfactorily.

### Computational Details

DFT calculations on compounds **4** to **6** were carried out using the GAUSSIAN09 package, the M06L functional, effective core potentials, and their associated double-ζ LANL2DZ basis set for metal atoms and 6-31G* basis for light elements (P, S, O, C, and H) as described previously.^5^ NMR shielding contributions were calculated using the gauge-including atomic orbitals method^42^ in combination with the LANL2DZ basis set for the metal atoms and the IGLO-III basis set of Kutzelnigg and co-workers for the remaining atoms.^43^

## Supporting Information Available

Crystal data for compounds **2**, **3b**, **4**, **5**, and ***syn***- and ***anti*-6** and **7a** (CCDC 2236982 and 2227092 to 2227097), IR and NMR spectra for all new compounds, and results of DFT calculations on compounds **4** to **6** (PDF).
Cartesian coordinates of all DFT-computed species (XYZ)

## Supplementary Material

ic3c00230_si_001.pdf

ic3c00230_si_002.xyz
