# Supplementary material for: Reactions of Heterometallic Phosphinidene-Bridged MoMn and MoRe Complexes with Sulfur and Selenium: From Chalcogenophosphinidene- to Trithiophosphonate-Bridged Derivatives
Source: Inorg Chem. 2023 Mar 29;62(14):5677–89. doi: 10.1021/acs.inorgchem.3c00230 (PMC10091403; doi:10.1021/acs.inorgchem.3c00230)
Supplement: Supplementary file 1 — ic3c00230_si_001.pdf [file ic3c00230_si_001.pdf]

Supporting Information

Reactions of Heterometallic Phosphinidene-Bridged MoMn and MoRe Complexes with Sulfur and Selenium: From Chalcogenophosphinidene- to Trithiophosphonate-Bridged Derivatives.

M. Angeles Alvarez, M. Esther García, Daniel García-Vivó,* Miguel A. Ruiz,* and Patricia Vega

Departamento de Química Orgánica e Inorgánica/IUQOEM, Universidad de Oviedo, E-33071 Oviedo, Spain.

Corresponding Author E-mail: garciavdaniel@uniovi.es (D.G.V.), mara@uniovi.es (M.A.R).

Table S1. Crystal Data for New Compounds (I)

	**2**	**3b**	**4**	**5**
mol formula	$C_{29}H_{34}MoO_6PReS$	$C_{28}H_{34}MnMoO_5PS$	$C_{28}H_{34}MoO_5PReS_2$	$C_{28}H_{34}MoO_5PReS_2$
mol wt	823.74	664.46	827.79	827.79
cryst syst	triclinic	orthorhombic	triclinic	triclinic
space group	$P-1$	$Pna2_1$	$P-1$	$P-1$
radiation (λ, Å)	0.71073	1.54184	1.54184	1.54184
a, Å	11.8177(4)	18.3450(3)	9.7867(3)	9.4398(4)
b, Å	12.0633(5)	10.69660(10)	11.0671(5)	9.6392(4)
c, Å	13.7970(5)	30.2718(4)	16.0912(7)	23.5325(8)
α, deg	79.931(2)	90	79.418(4)	88.016(3)
β, deg	84.510(2)	90	79.163(3)	87.162(3)
γ, deg	86.781(2)	90	86.915(3)	69.012(4)
V, Å^3	1926.17(13)	5940.21(14)	1682.31(12)	1996.4(2)
Z	2	8	2	2
calcd density, g cm^{-3}	1.420	1.486	1.634	1.377
absorp coeff, mm^{-1}	3.592	8.365	11.850	9.986
temperature, K	100.0(1)	150.1(1)	150.4(6)	154(7)
θ range (deg)	1.72/31.51	2.92/69.54	4.06/69.45	3.76 /69.62
index ranges (h, k, l)	−17, 17; −17, 17 −20, 20	−22, 20; −12, 10 −35, 36	−9, 11; −13, 10 −19, 19	−8, 11; −11, 11 −27, 28
no. of reflns collected	156069	17286	13806	19353
no. of indep reflns (R_{int})	12841(0.0623)	8192 (0.0311)	6176 (0.0502)	7409 (0.0407)
reflns with $I > 2\sigma(I)$	10888	7715	5458	6608
R indexes [data with $I > 2\sigma(I)$][a]	R_1 = 0.0256 wR_2 = 0.0585[b]	R_1 = 0.0342 wR_2 = 0.0927[c]	R_1 = 0.0343 wR_2 = 0.0845[d]	R_1 = 0.0607 wR_2 = 0.1719[e]
R indexes (all data)[a]	R_1 = 0.0385 wR_2 = 0.0663[b]	R_1 = 0.0374 wR_2 = 0.0948[c]	R_1 = 0.0405 wR_2 = 0.0887[d]	R_1 = 0.0650 wR_2 = 0.1770[e]
GOF	1.065	1.100	1.029	1.057
no. of restraints/params	0 / 352	1 / 683	3 / 350	0 / 352
$\Delta\rho$(max., min.), eÅ^{-3}	1.811 / −1.383	0.666 / −0.755	1.310 / −1.854	3.879 / −2.465
CCDC deposition no	2236982	2227092	2227093	2227094

[a] $R = \Sigma||F_o| - |F_c|| / \Sigma|F_o|$. $wR = [\Sigma w(|F_o|^2 - |F_c|^2)^2 / \Sigma w|F_o|^2]^{1/2}$. $w = 1/[\sigma^2(F_o^2) + (aP)^2 + bP]$ where $P = (F_o^2 + 2F_c^2)/3$. [b] a = 0.0290, b = 2.6483. [c] a = 0.0478, b = 5.7993. [d] a = 0.0447, b = 0.2570. [e] a = 0.1156, b = 4.6194.

Table S2. Crystal Data for New Compounds (II)

	***syn*-6**·CH_2Cl_2	***anti*-6**·1/2CH_2Cl_2	**7a**
mol formula	$C_{29}H_{36}Cl_2MoO_5PReS_3$	$C_{57}H_{70}Cl_2Mo_2O_{10}P_2Re_2S_6$	$C_{28}H_{34}MoO_5PReSe$
mol wt	944.77	1804.63	842.62
cryst syst	triclinic	triclinic	orthorhombic
space group	*P*–1	*P*–1	$Pna2_1$
radiation (λ, Å)	0.71073	1.54184	1.54184
a, Å	9.4955(3)	13.6067(3)	18.3290(3)
b, Å	13.0426(5)	15.4497(3)	10.7749(2
c, Å	14.0622(5)	34.0149(4)	30.4775(6)
α, deg	96.210(1)	77.3750(10)	90
β, deg	100.882(1)	79.9490(10)	90
γ, deg	92.089(1)	74.766(2)	90
V, $Å^3$	1697.3(1)	6680.2(2)	6019.10(19)
Z	2	4	8
calcd density, g cm^{-3}	1.849	1.794	1.860
absorp coeff, mm^{-1}	4.358	13.285	13.323
temperature, K	100.0(1)	153(5)	151(1)
θ range (deg)	2.19/28.28	3.02/69.63	2.90 /69.58
index ranges (*h*, *k*, *l*)	–12, 12; –17, 17 –18, 18	–15, 16; –18, 18 –40, 38	–21, 22; –12, 12 –36, 34
no. of reflns collected	81006	73910	19859
no. of indep reflns (R_{int})	8423 (0.0332)	24858 (0.0410)	8511 (0.0515)
reflns with $I > 2\sigma(I)$	8128	23010	7574
R indexes [data with $I > 2\sigma(I)$][a]	R_1 = 0.0160 wR_2 = 0.0387[b]	R_1 = 0.0319 wR_2 = 0.0920[c]	R_1 = 0.0496 wR_2 = 0.1326[d]
R indexes (all data)[a]	R_1 = 0.0174 wR_2 = 0.0393[b]	R_1 = 0.0342 wR_2 = 0.0942[c]	R_1 = 0.0574 wR_2 = 0.1460[d]
GOF	1.113	1.083	1.061
no. of restraints/params	0 / 388	0 / 443	25 / 666
$\Delta\rho$(max., min.), e$Å^{-3}$	0.942 / –0.565	1.019 / –1.590	1.897 / –1.987
CCDC deposition no	2227095	2227096	2227097

[a] $R = \Sigma||F_o| - |F_c|| / \Sigma|F_o|$. $wR = [\Sigma w(|F_o|^2 - |F_c|^2)^2 / \Sigma w|F_o|^2]^{1/2}$. $w = 1/[\sigma^2(F_o^2) + (aP)^2 + bP]$ where $P = (F_o^2 + 2F_c^2)/3$. [b] *a* = 0.0153, *b* = 1.8564. [c] *a* = 0.0596, *b* = 0.0000. [d] *a* = 0.0792, *b* = 25.4999.

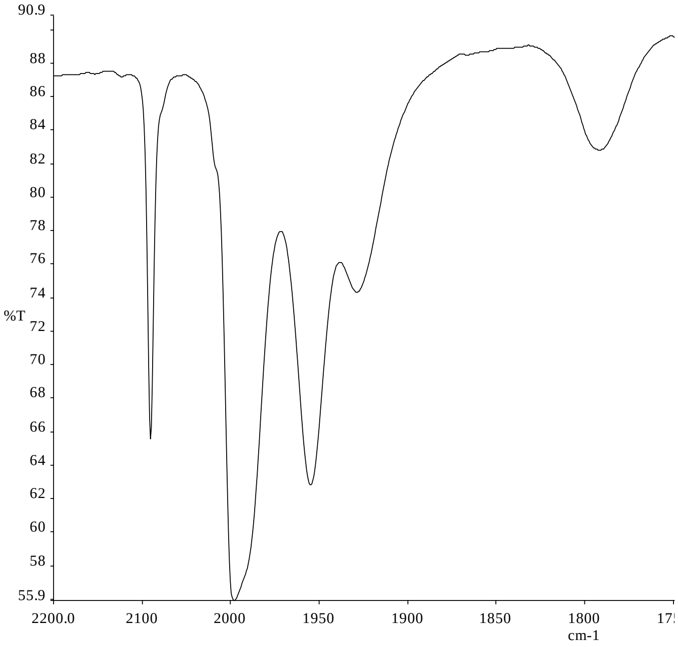

Figure S1. IR spectrum of compound **2** in dichloromethane solution.

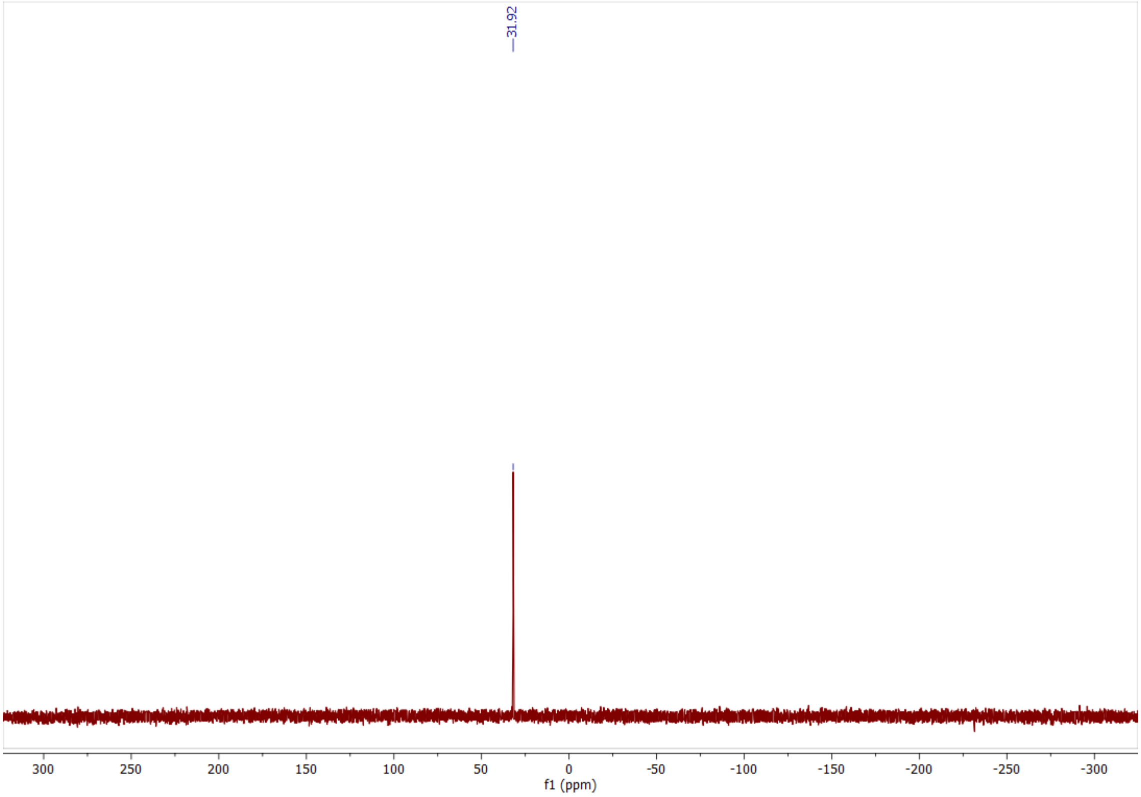

Figure S2. $^{31}P\{^{1}H\}$ NMR spectrum of compound **2** ($CDCl_2$).

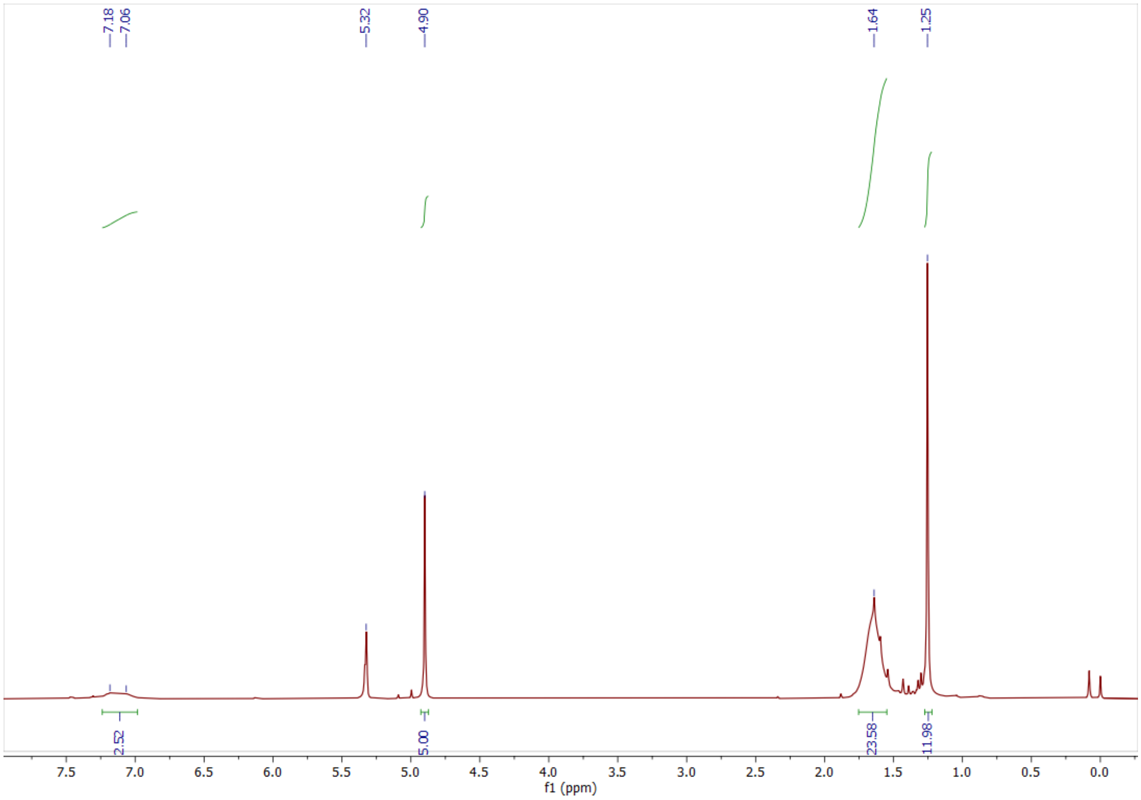

Figure S3. ^{1}H NMR spectrum of compound **2** (CD_2Cl_2).

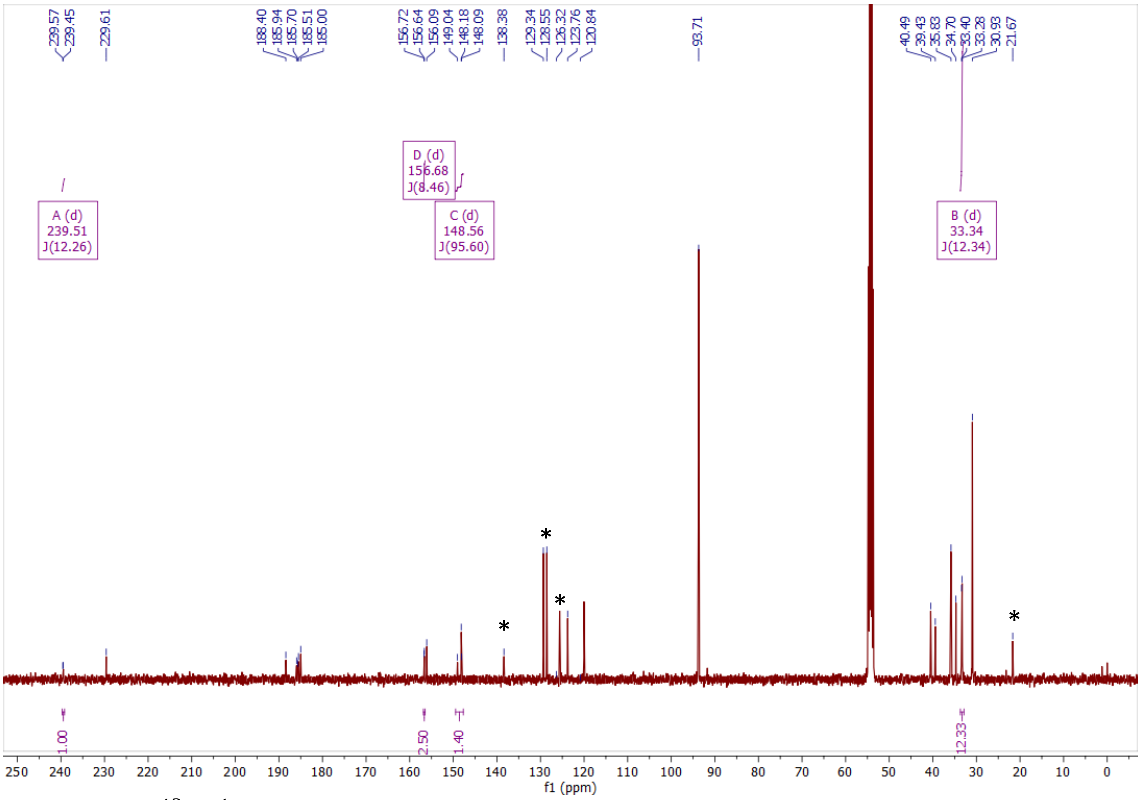

Figure S4. ^{13}C{^{1}H} NMR spectrum of compound **2** (CD_2Cl_2, 243 K). Resonances marked with * correspond to residual toluene in the sample.

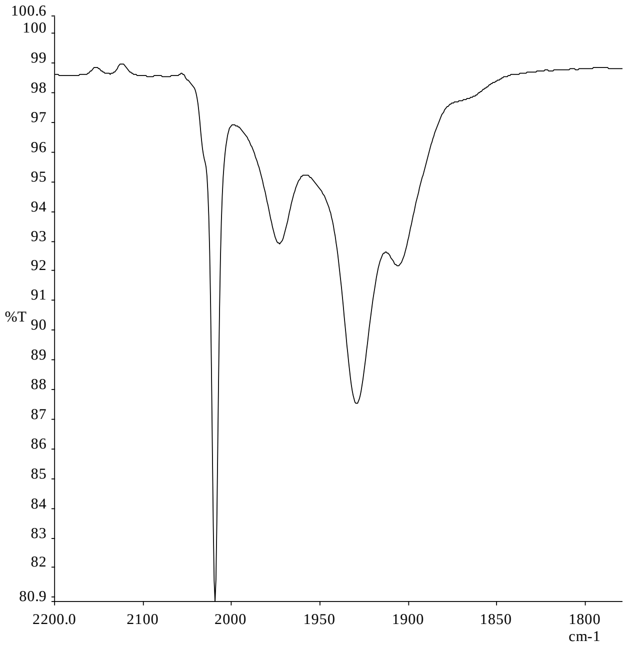

Figure S5. IR spectrum of compound **3a** in dichloromethane solution.

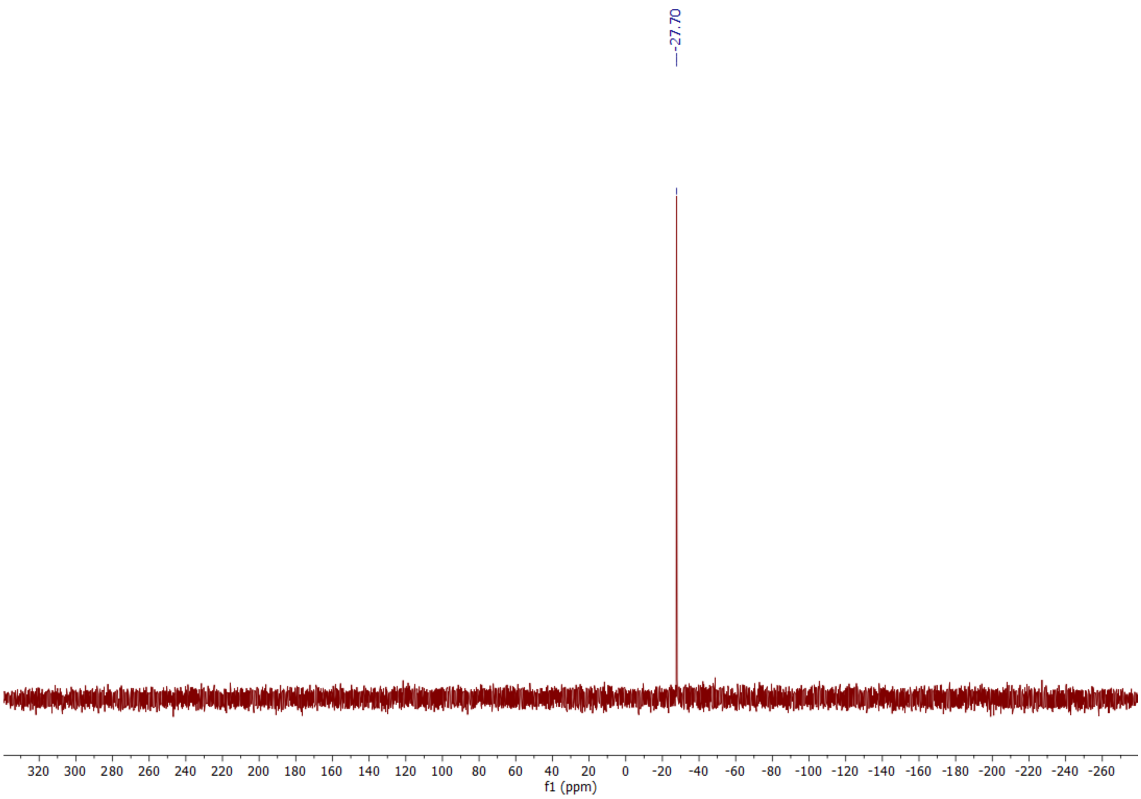

Figure S6. $^{31}P\{^{1}H\}$ NMR spectrum of compound **3a** (CD_2Cl_2).

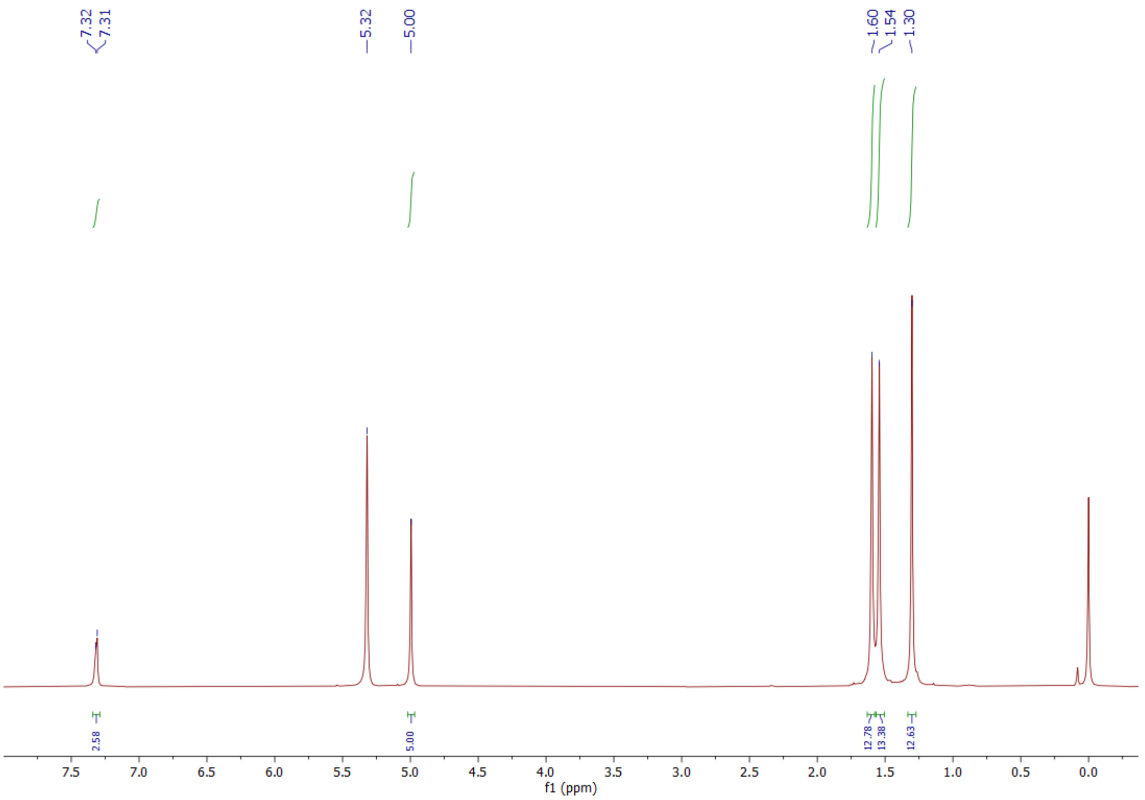

Figure S7. ^{1}H NMR spectrum of compound **3a** (CD_2Cl_2).

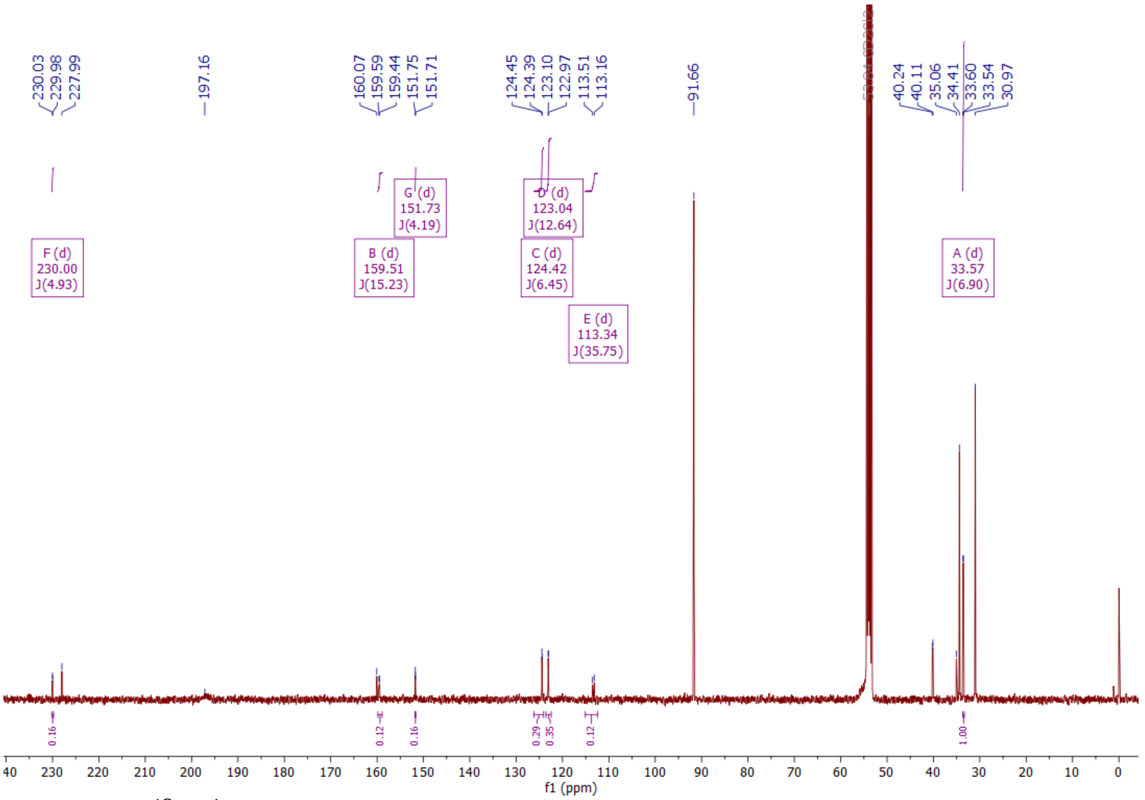

Figure S8. $^{13}C\{^{1}H\}$ NMR spectrum of compound **3a** (CD_2Cl_2).

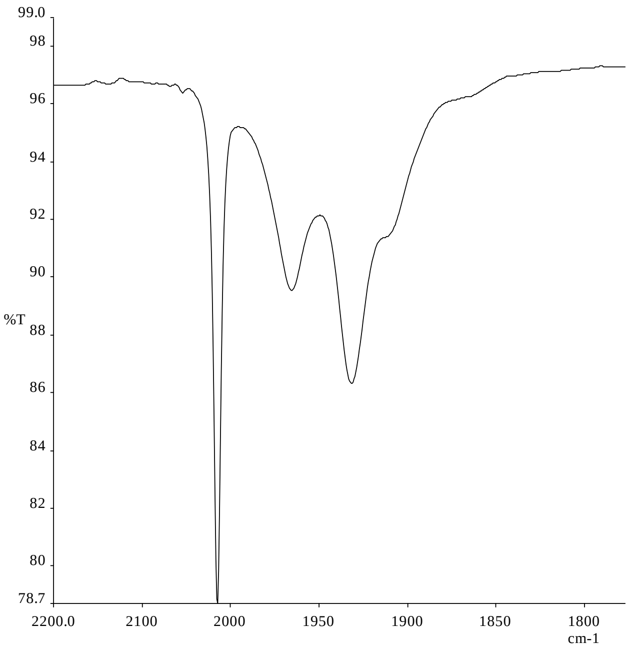

Figure S9. IR spectrum of compound **3b** in dichloromethane solution.

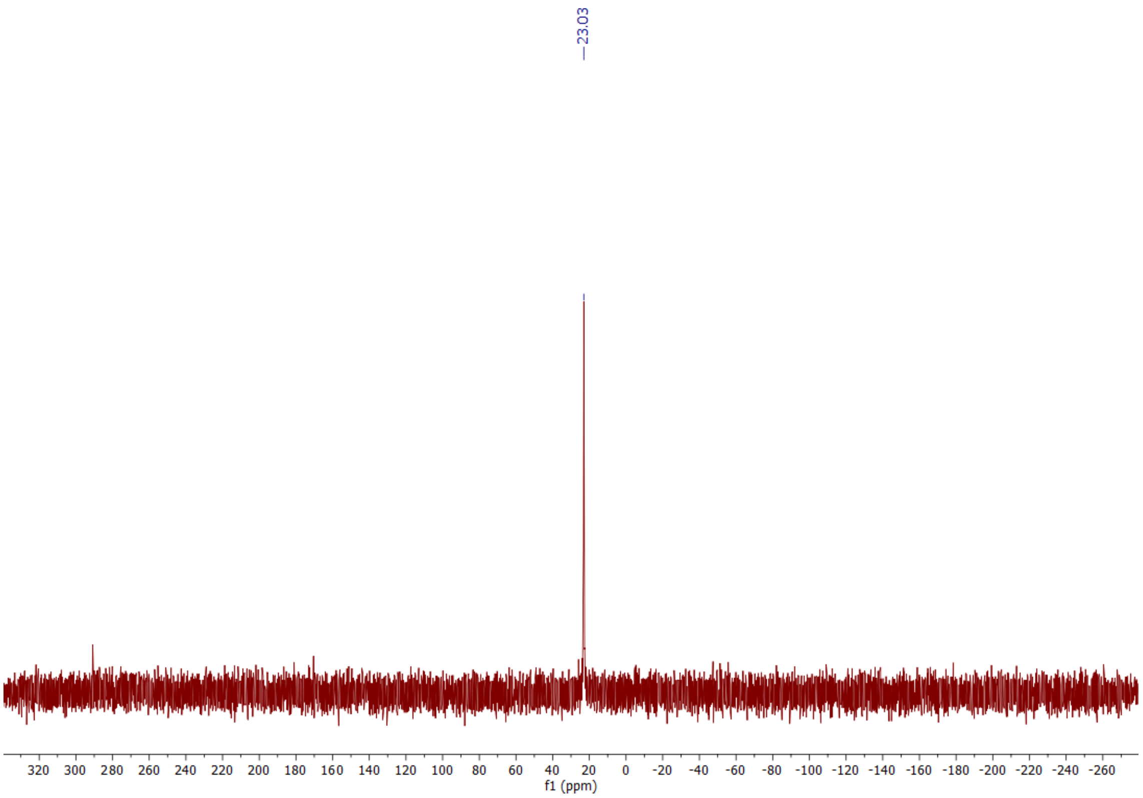

Figure S10. $^{31}P\{^{1}H\}$ NMR spectrum of compound **3b** (CD_2Cl_2).

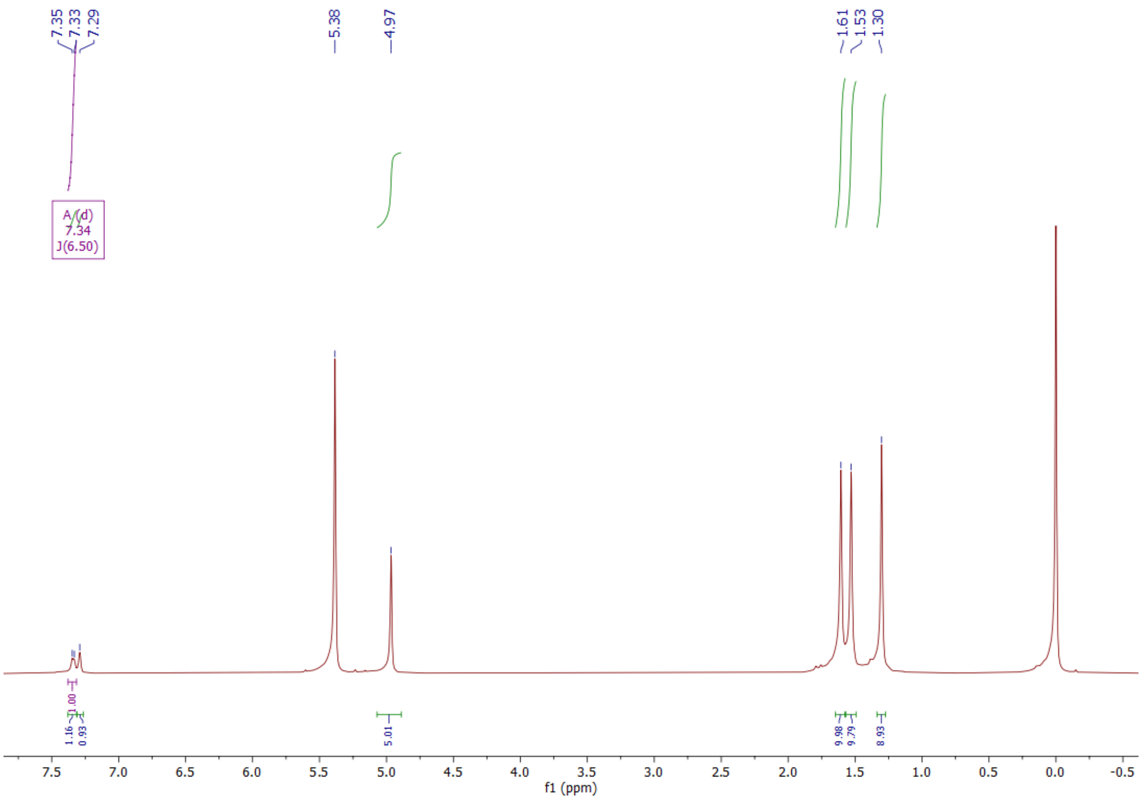

Figure S11. ^{1}H NMR spectrum of compound **3b** (CD_2Cl_2, 233 K).

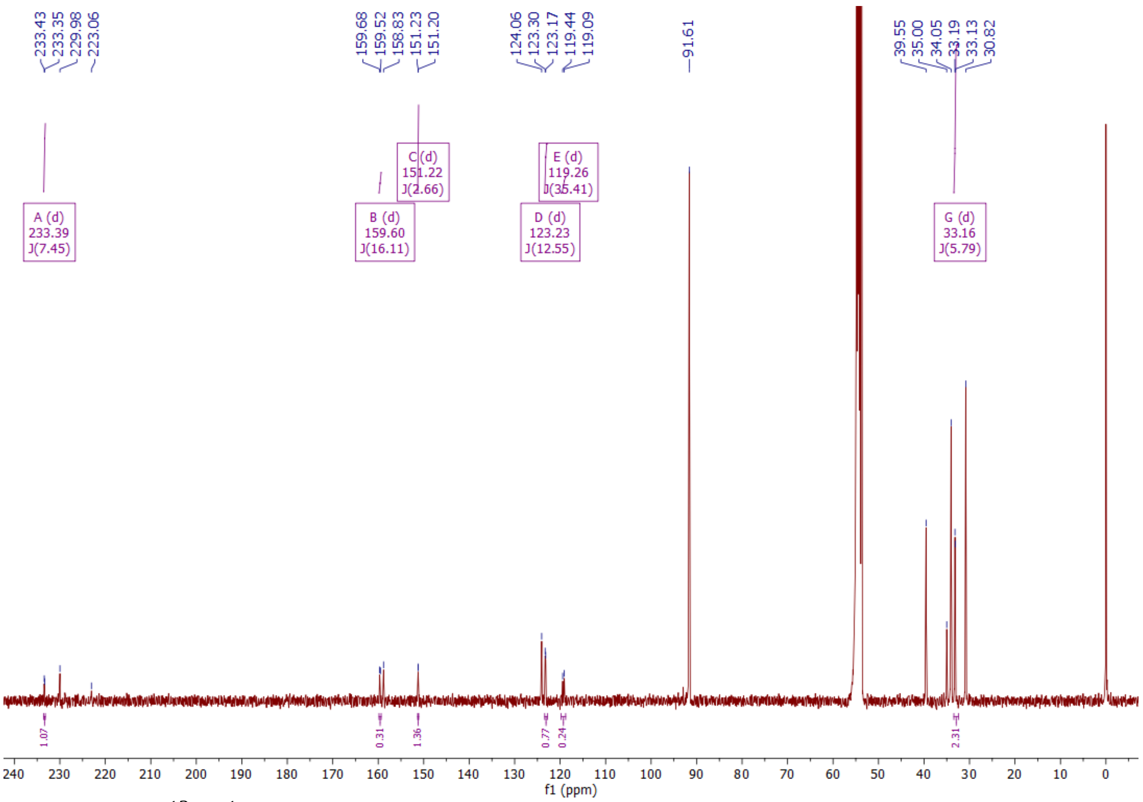

Figure S12. $^{13}C\{^{1}H\}$ NMR spectrum of compound **3b** (CD_2Cl_2, 233 K).

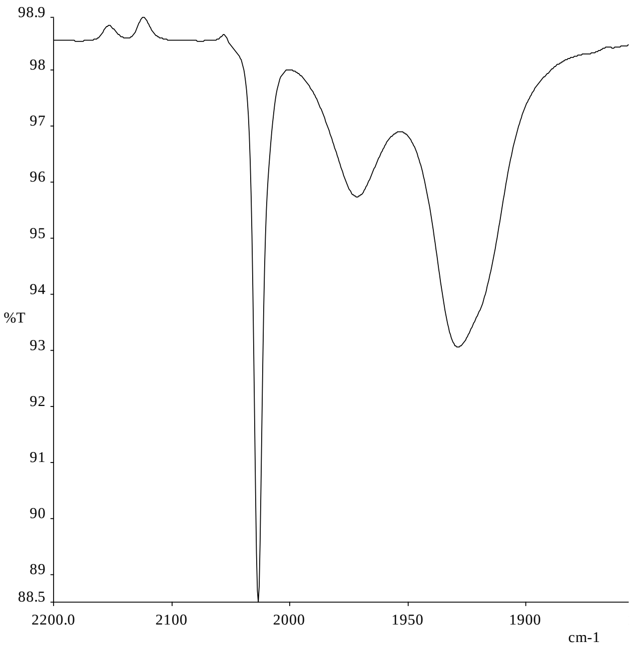

Figure S13. IR spectrum of compound **4** in dichloromethane solution.

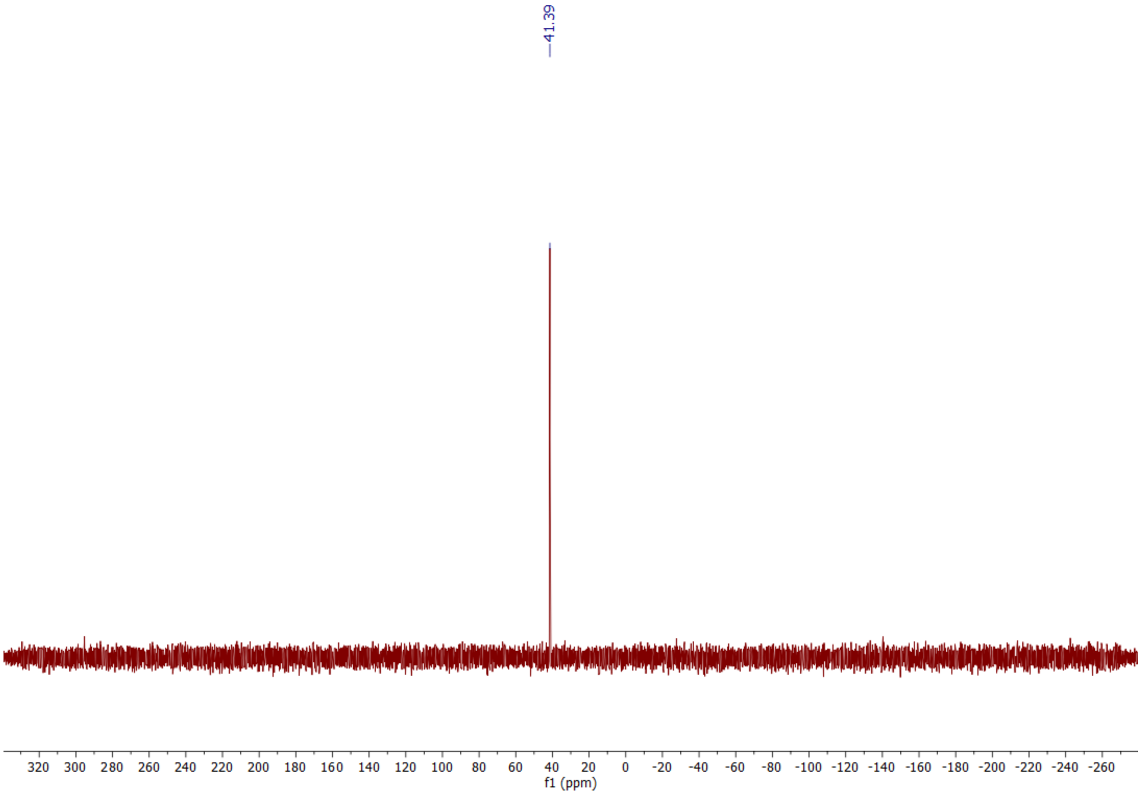

Figure S14. $^{31}P\{^1H\}$ NMR spectrum of compound **4** (CD_2Cl_2).

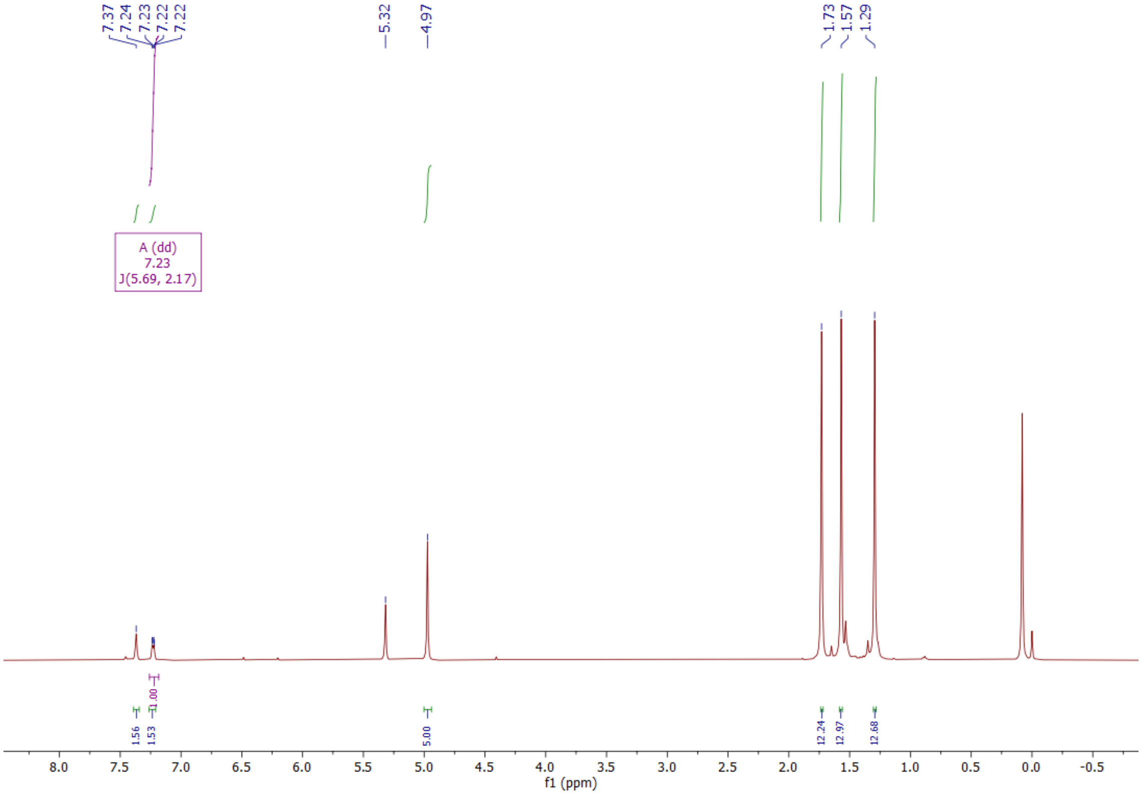

Figure S15. ^{1}H NMR spectrum of compound **4** (CD_2Cl_2).

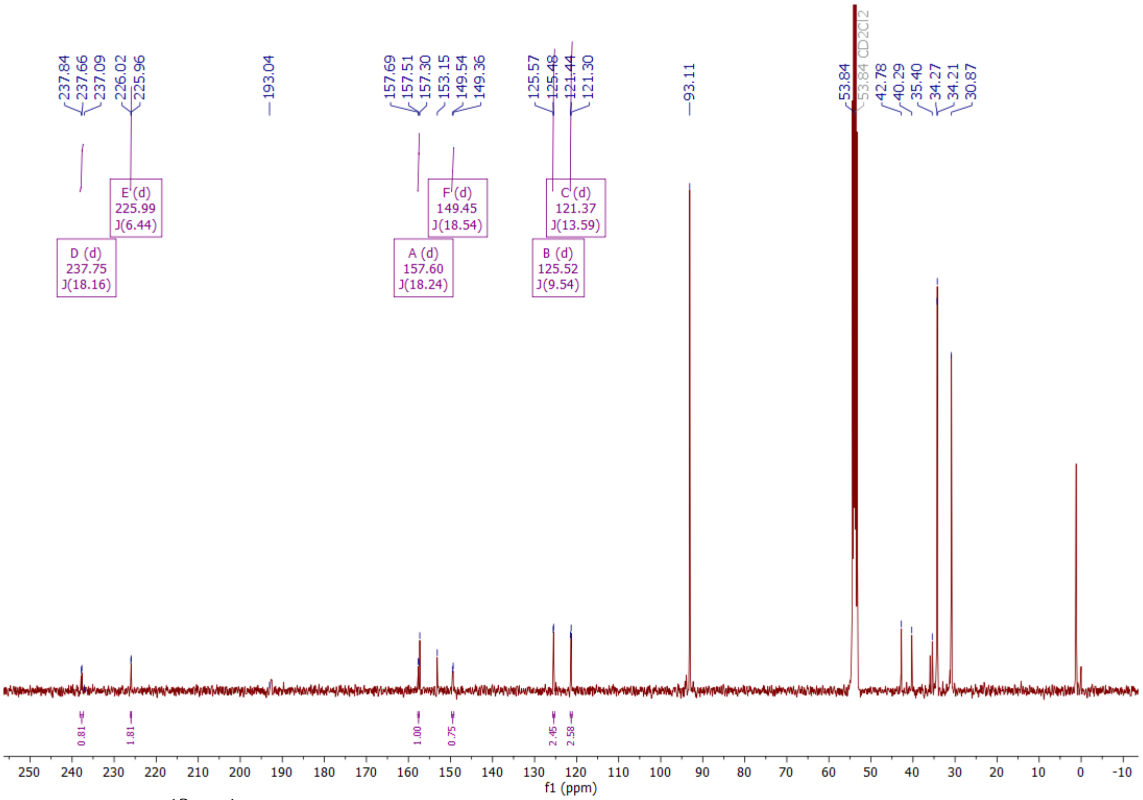

Figure S16. $^{13}C\{^{1}H\}$ NMR spectrum of compound **4** (CD_2Cl_2).

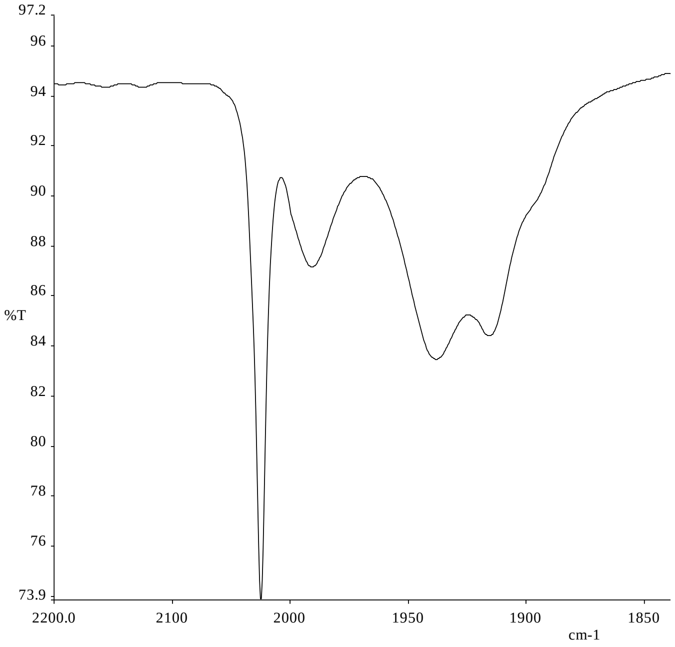

Figure S17. IR spectrum of compound **5** in dichloromethane solution.

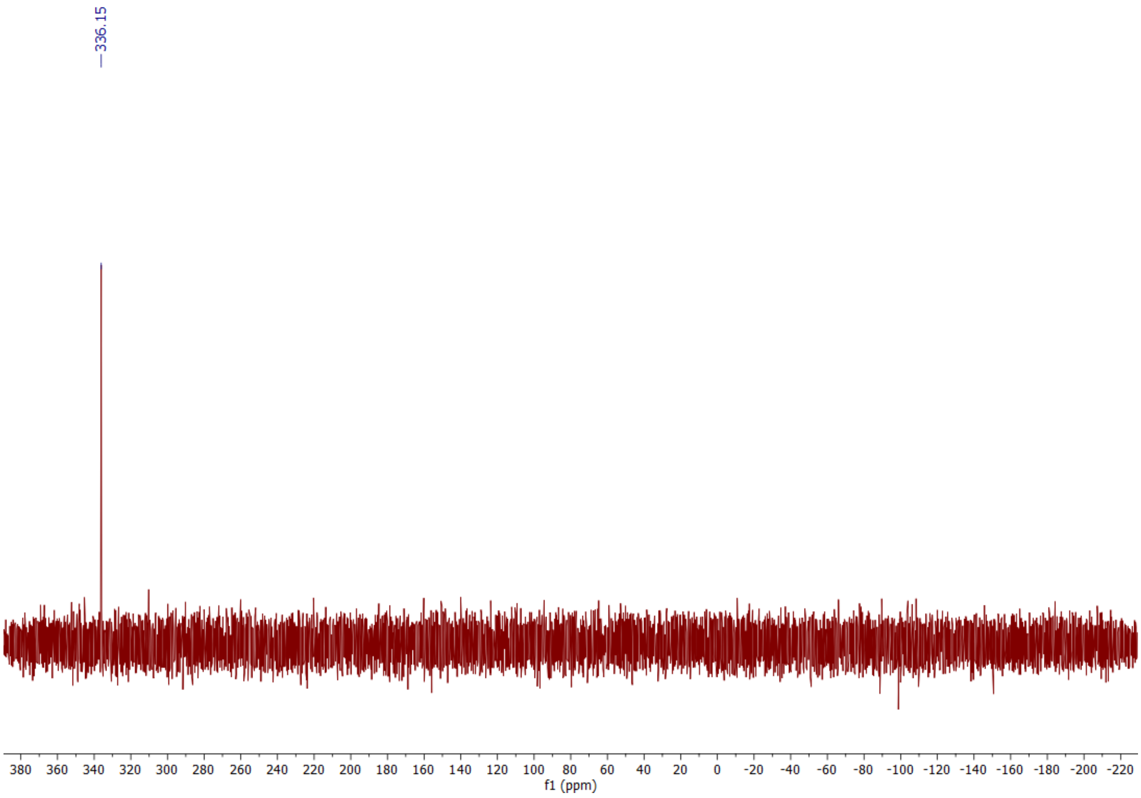

Figure S18. $^{31}P\{^1H\}$ NMR spectrum of compound **5** (CD_2Cl_2).

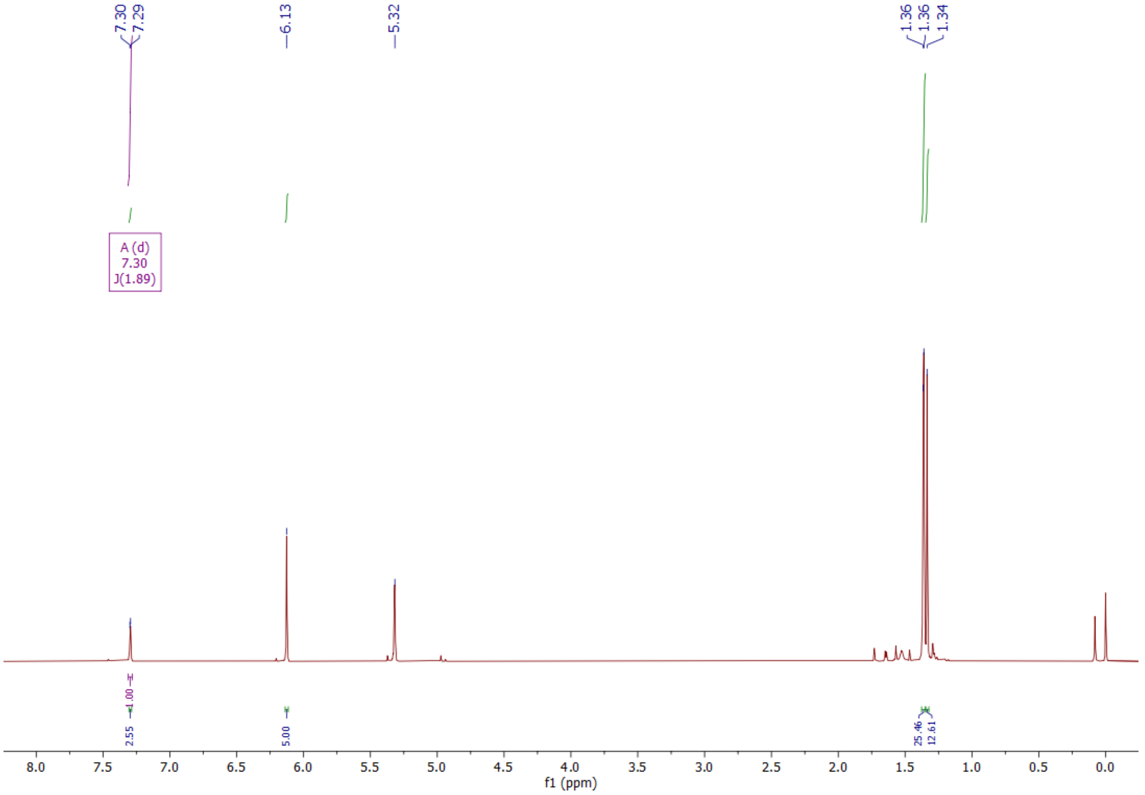

Figure S19. ^{1}H NMR spectrum of compound **5** (CD_2Cl_2).

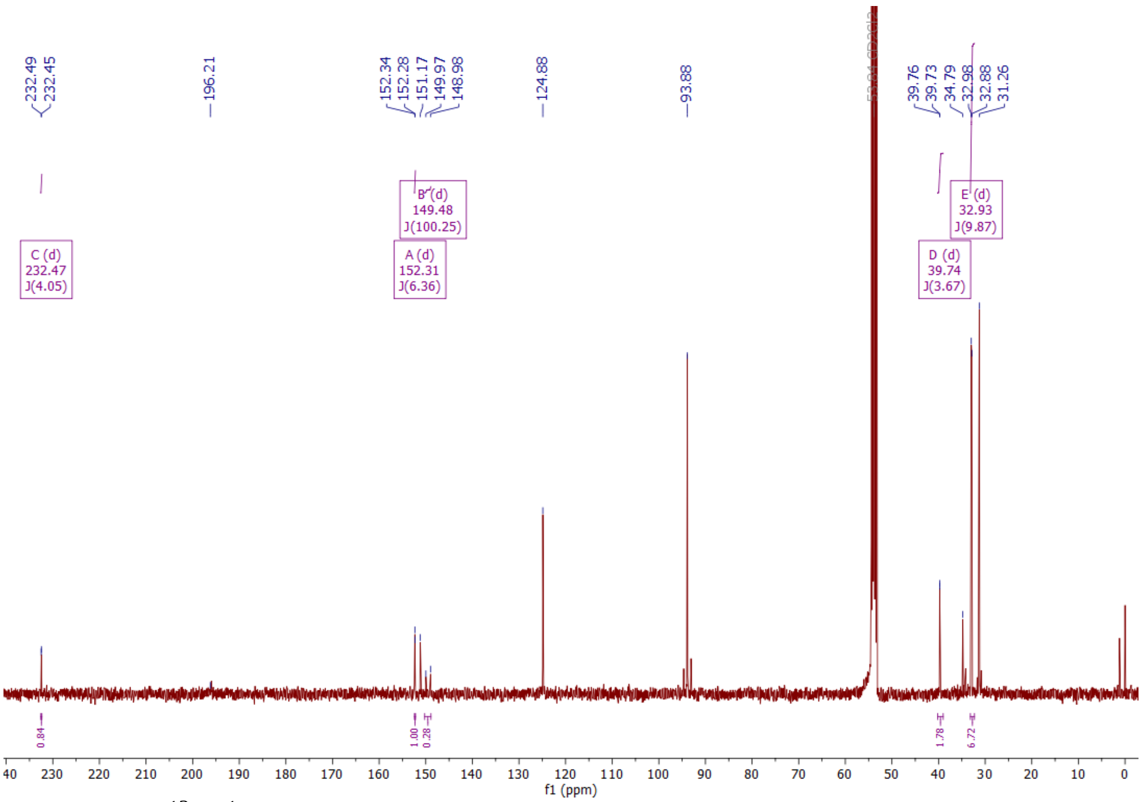

Figure S20. $^{13}C\{^{1}H\}$ NMR spectrum of compound **5** (CD_2Cl_2).

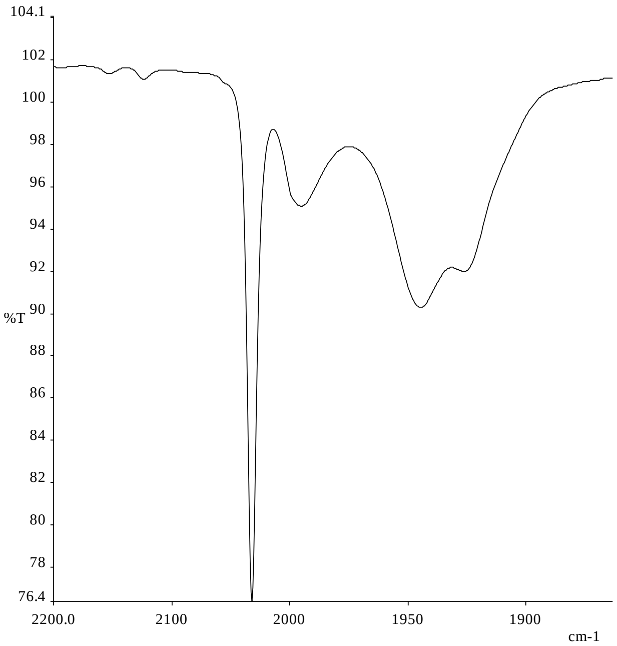

Figure S21. IR spectrum of compound ***syn*-6** in dichloromethane solution.

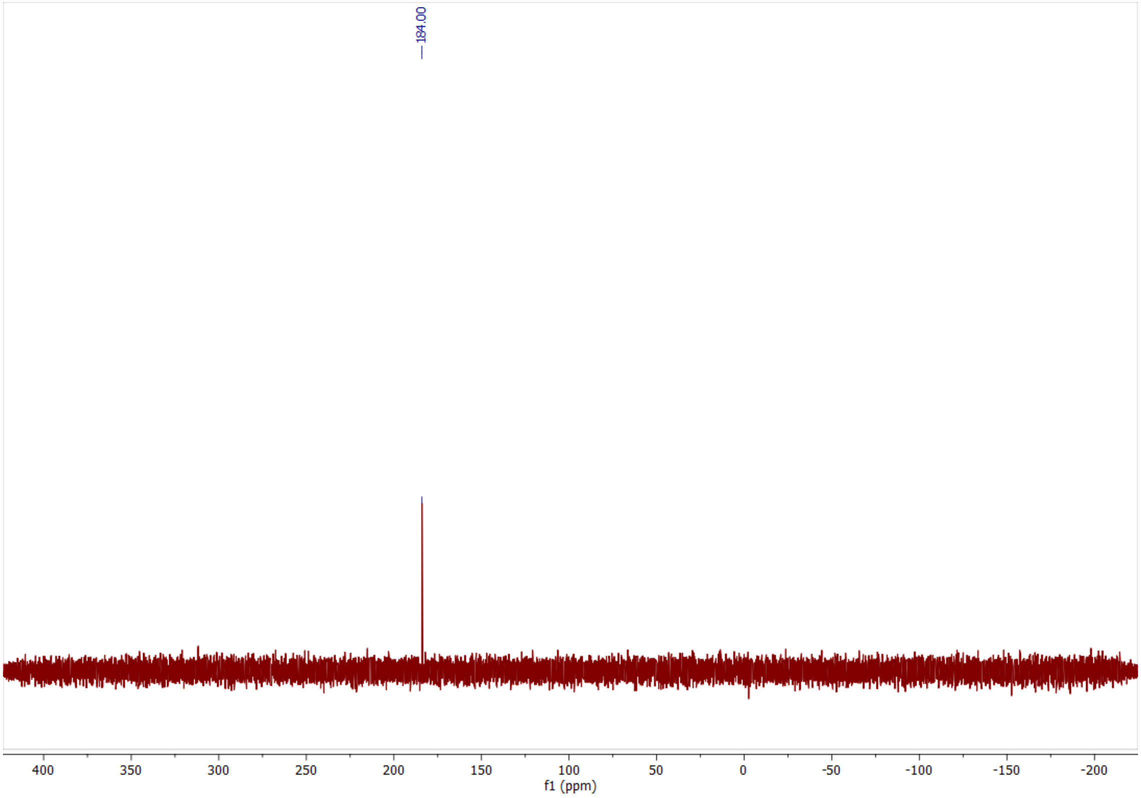

Figure S22. $^{31}P\{^{1}H\}$ NMR spectrum of compound ***syn*-6** (CD_2Cl_2).

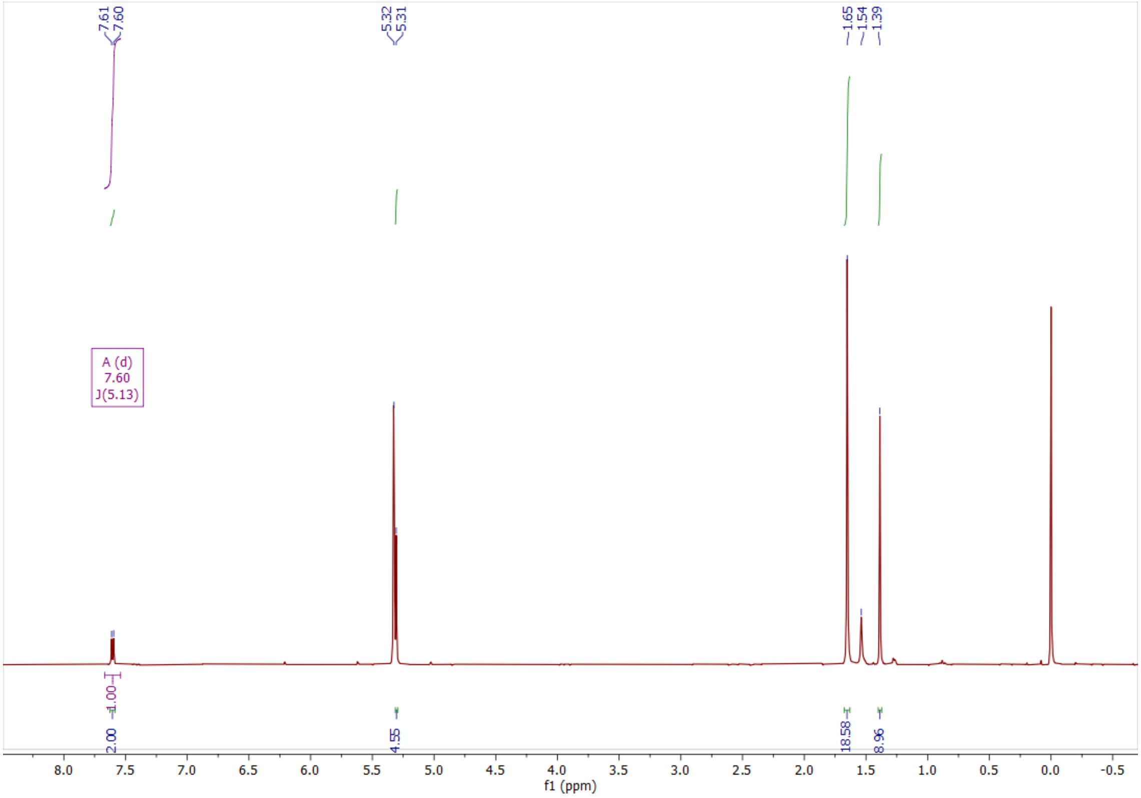

Figure S23. ^{1}H NMR spectrum of compound ***syn*-6** (CD_2Cl_2).

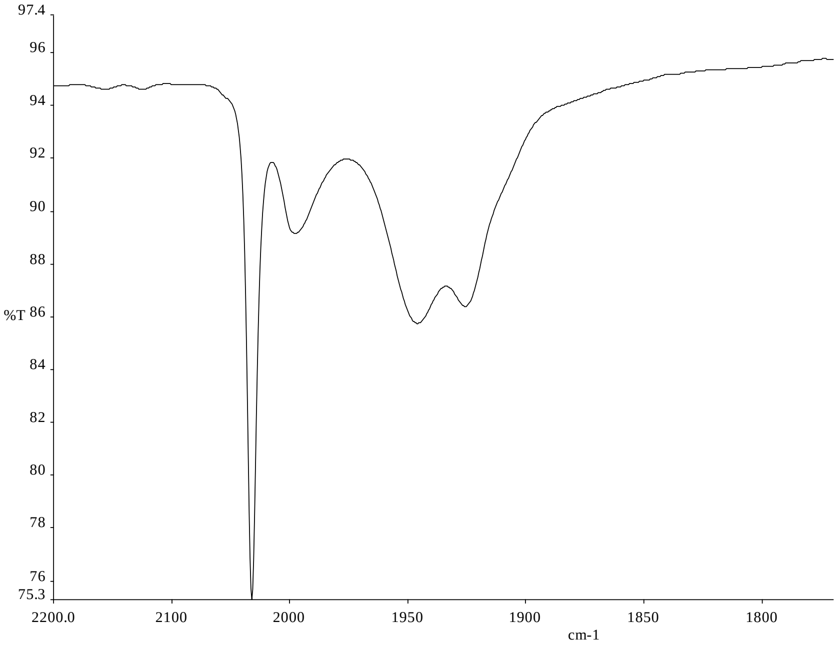

Figure S24. IR spectrum of compound ***anti*-6** in dichloromethane solution.

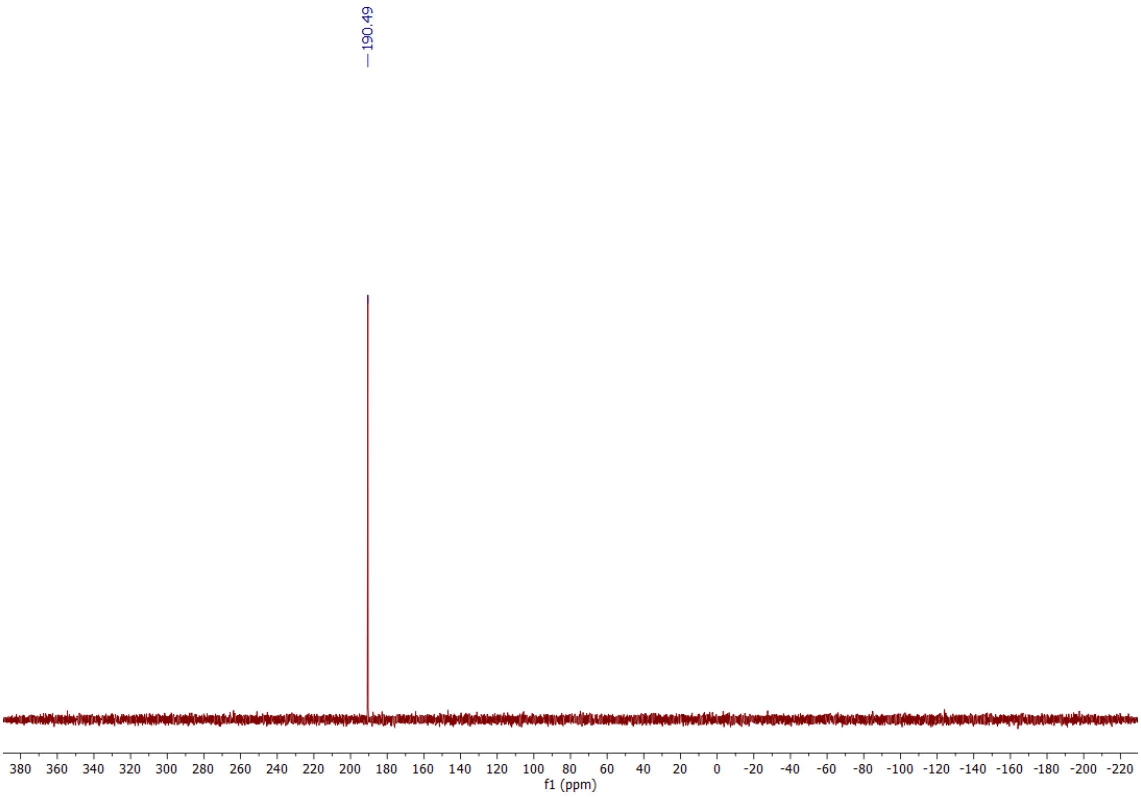

Figure S25. $^{31}P\{^1H\}$ NMR spectrum of compound ***anti*-6** (CD_2Cl_2).

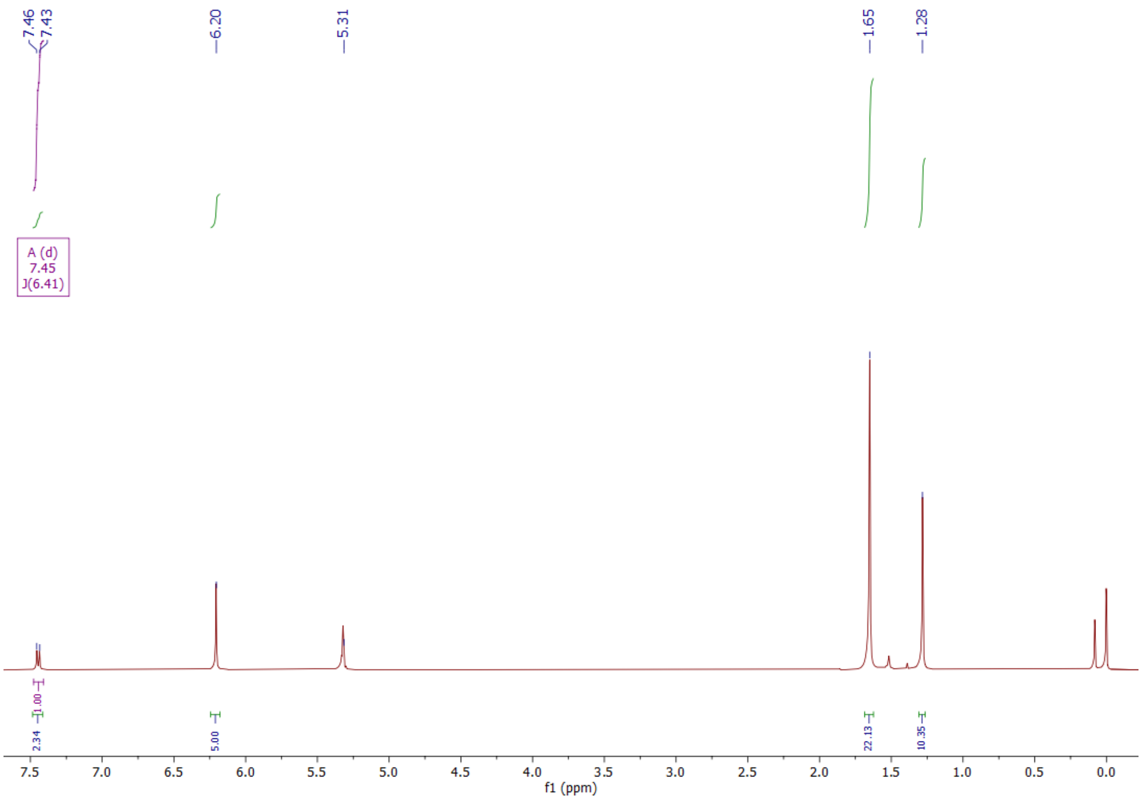

Figure S26. ^{1}H NMR spectrum of compound ***anti*-6** (CD_2Cl_2).

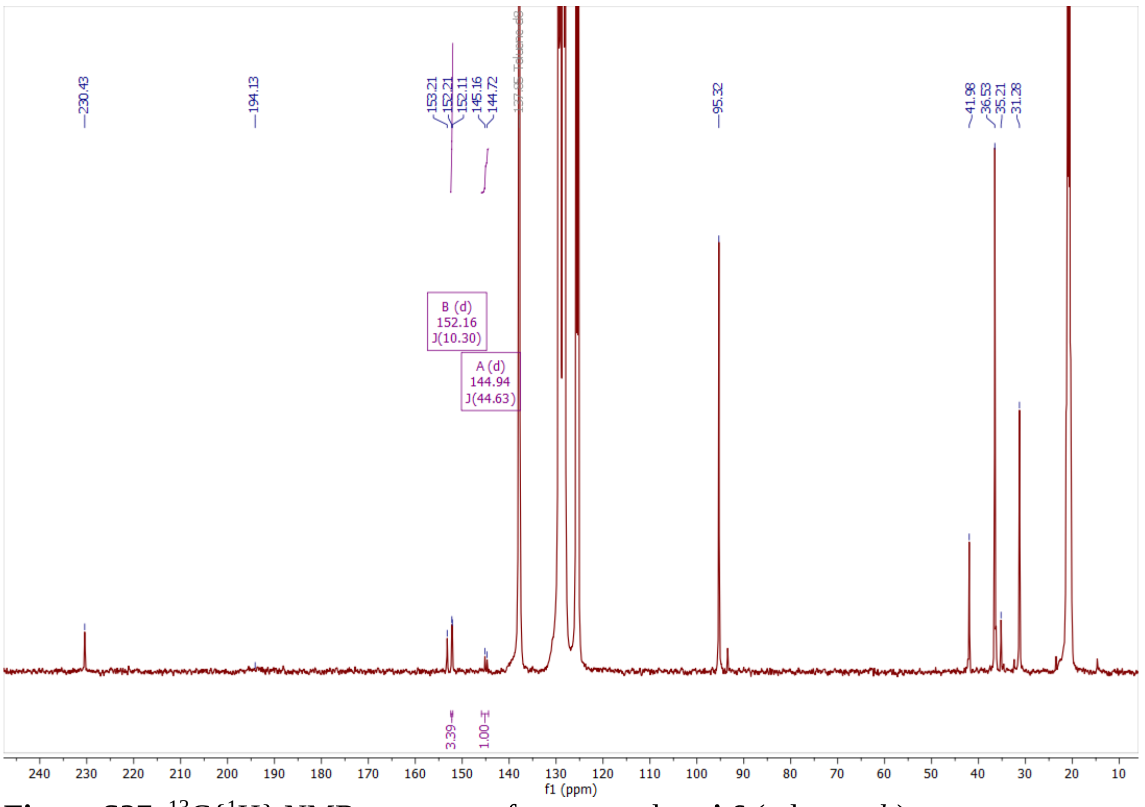

Figure S27. $^{13}C\{^{1}H\}$ NMR spectrum of compound ***anti*-6** (toluene-d_8).

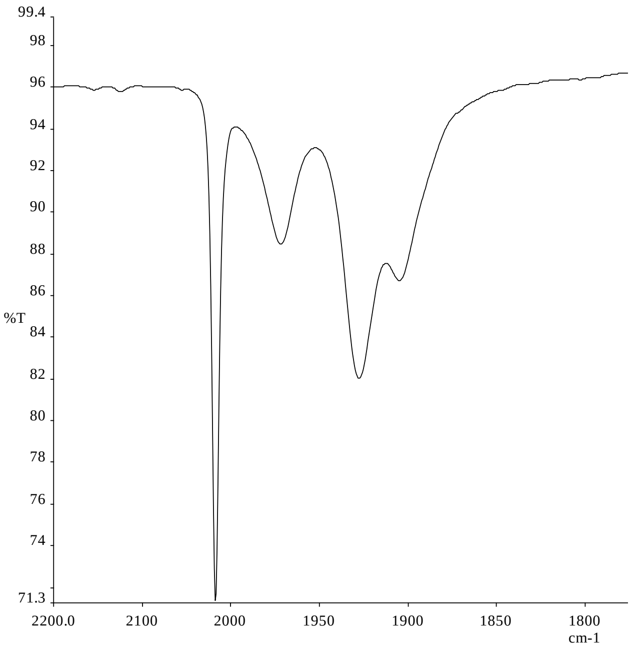

Figure S28. IR spectrum of compound **7a** in dichloromethane solution.

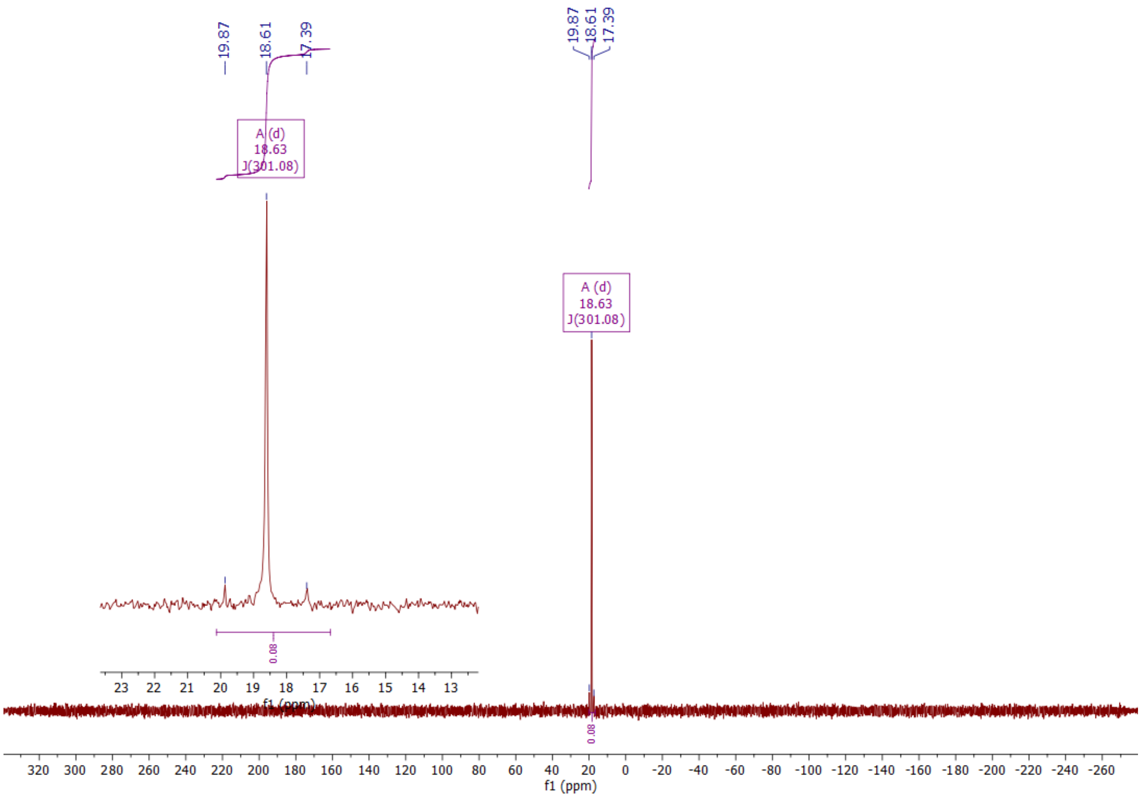

Figure S29. $^{31}P\{^{1}H\}$ NMR spectrum of compound **7a** (CD_2Cl_2).

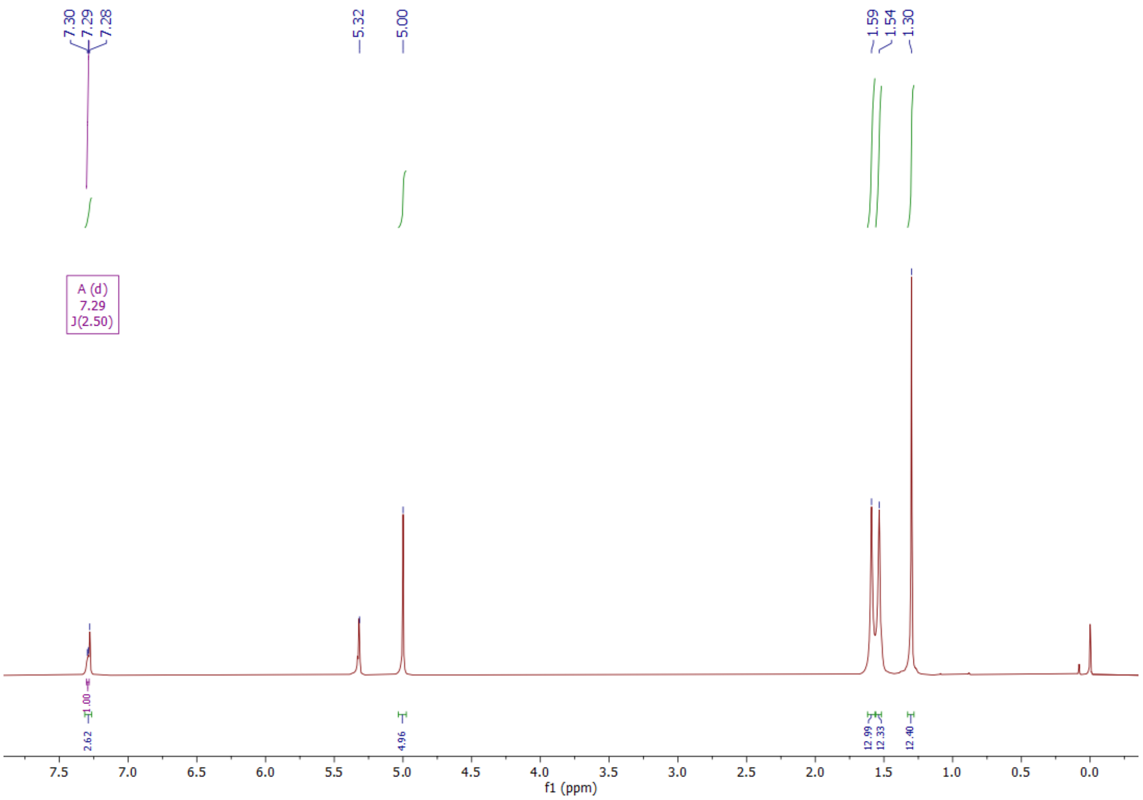

Figure S30. ^{1}H NMR spectrum of compound **7a** (CD_2Cl_2).

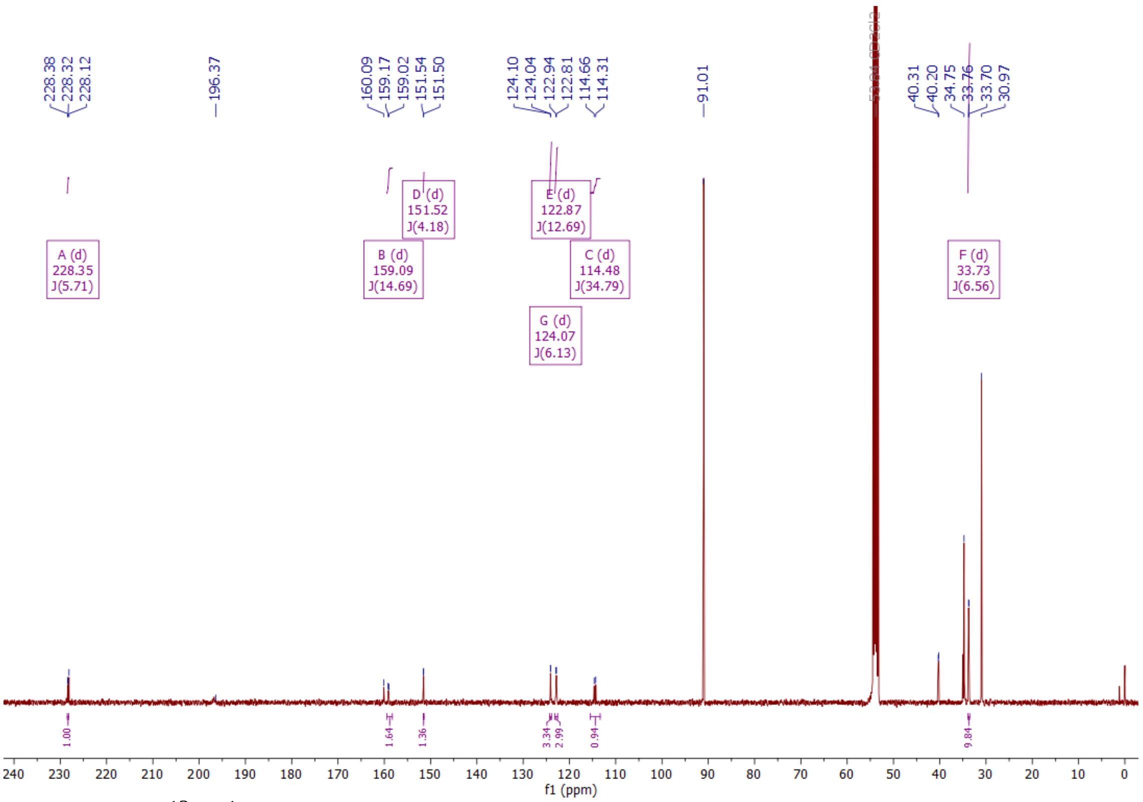

Figure S31. $^{13}C\{^{1}H\}$ NMR spectrum of compound **7a** (CD_2Cl_2).

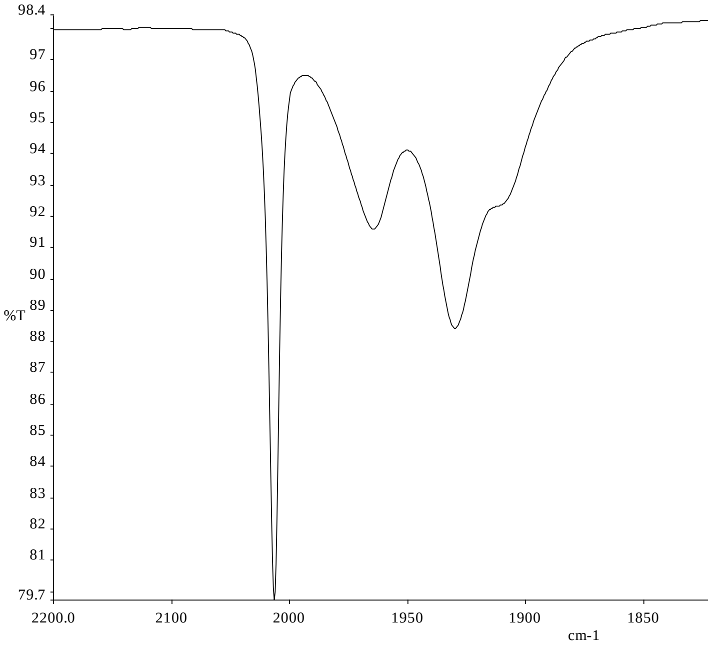

Figure S32. IR spectrum of compound **7b** in dichloromethane solution.

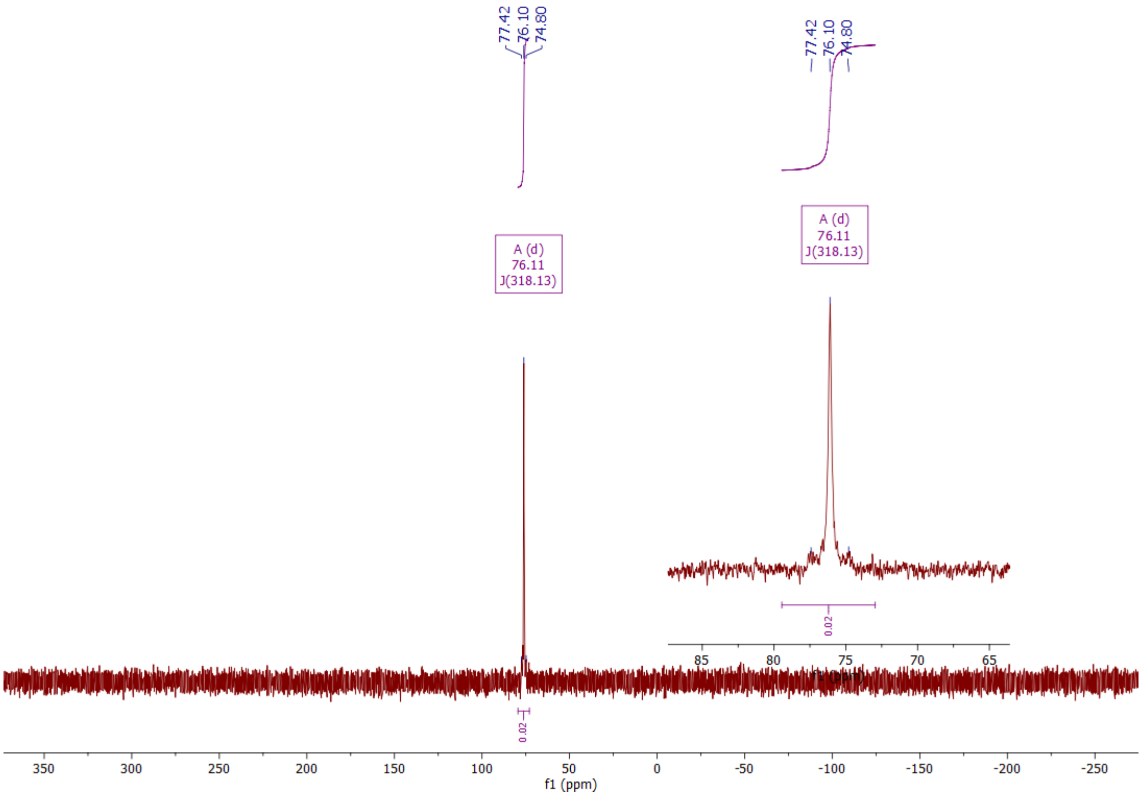

Figure S33. $^{31}P\{^{1}H\}$ NMR spectrum of compound **7b** (CD_2Cl_2).

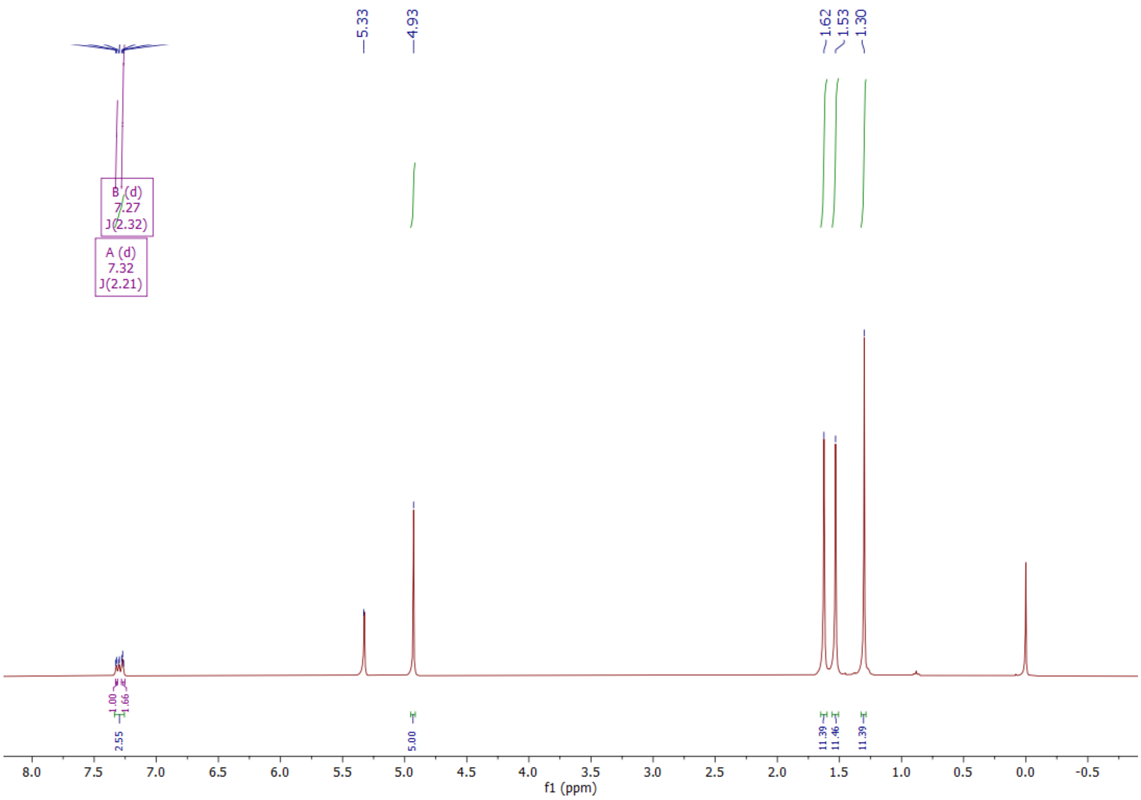

Figure S34. ^{1}H NMR spectrum of compound **7b** (CD_2Cl_2).

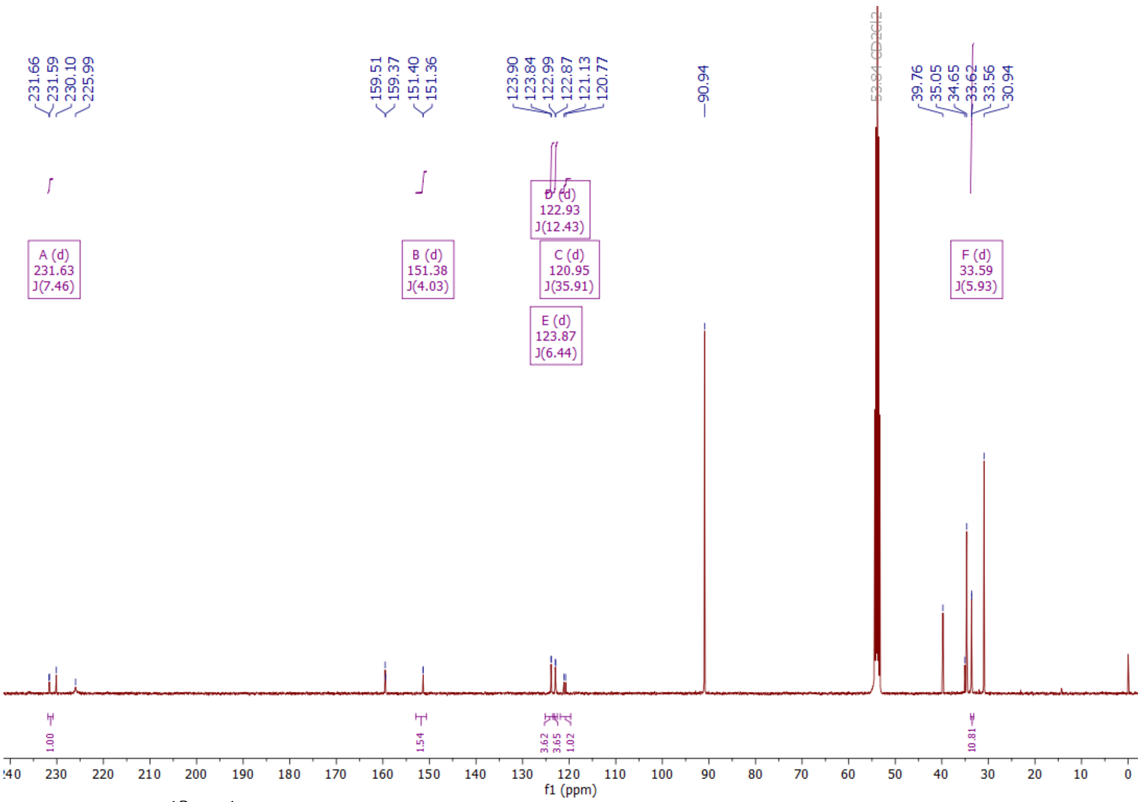

Figure S35. $^{13}C\{^{1}H\}$ NMR spectrum of compound **7b** (CD_2Cl_2).

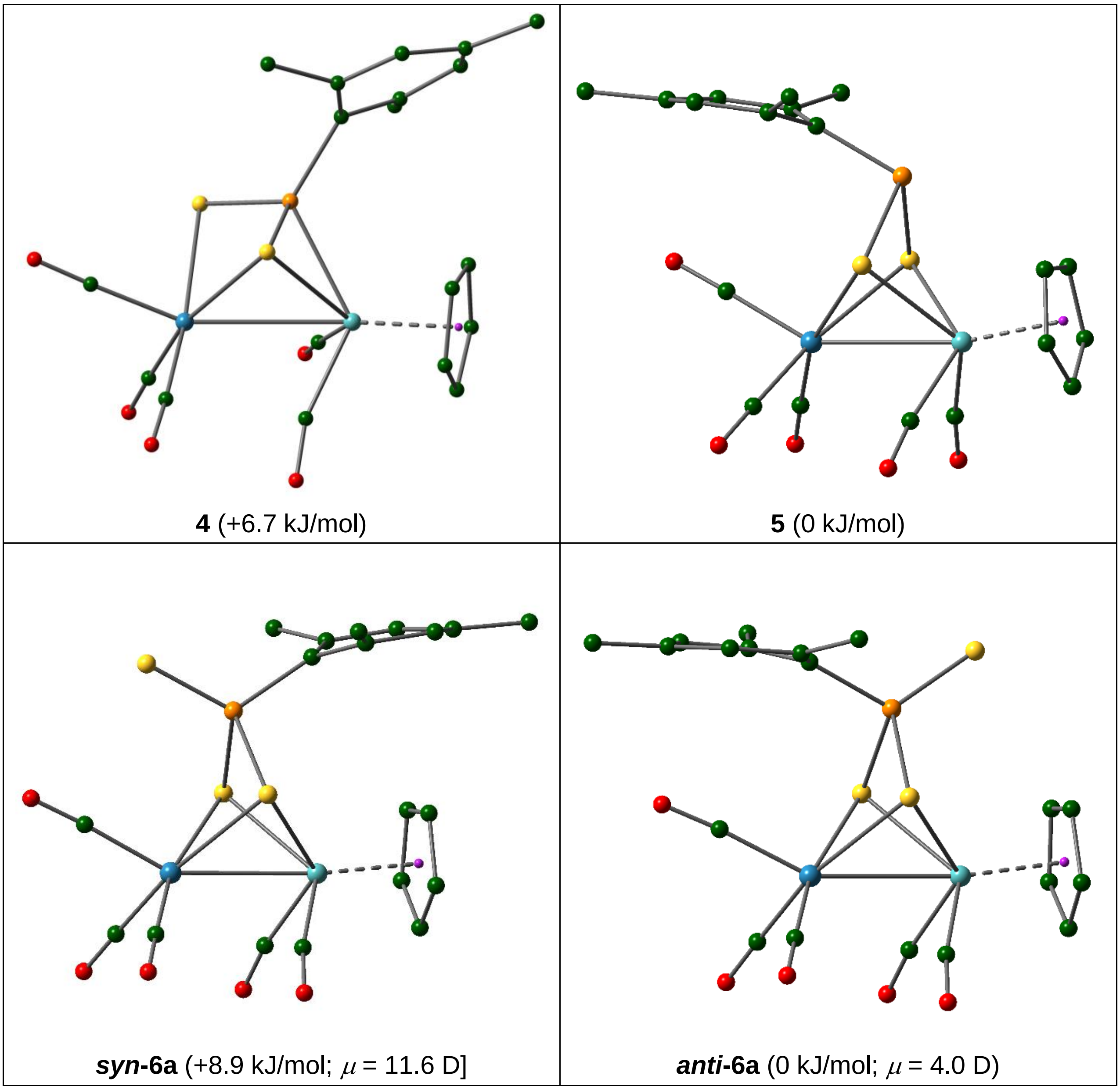

Figure S36. M06L-DFT optimized structures of compounds **4** to **6**, with relative Gibbs free energies (in kJ/mol) and dipolar moments (μ, in Debye) indicated below, in its case.
